# Supplementary material for: Structural insights into tRNA recognition of the human FTSJ1-THADA complex
Source: Commun Biol. 2025 Jun 7;8:893. doi: 10.1038/s42003-025-08278-3 (PMC12145424; doi:10.1038/s42003-025-08278-3)
Supplement: Supplementary file 2 — Supplementary_information [file 42003_2025_8278_MOESM2_ESM.pdf]

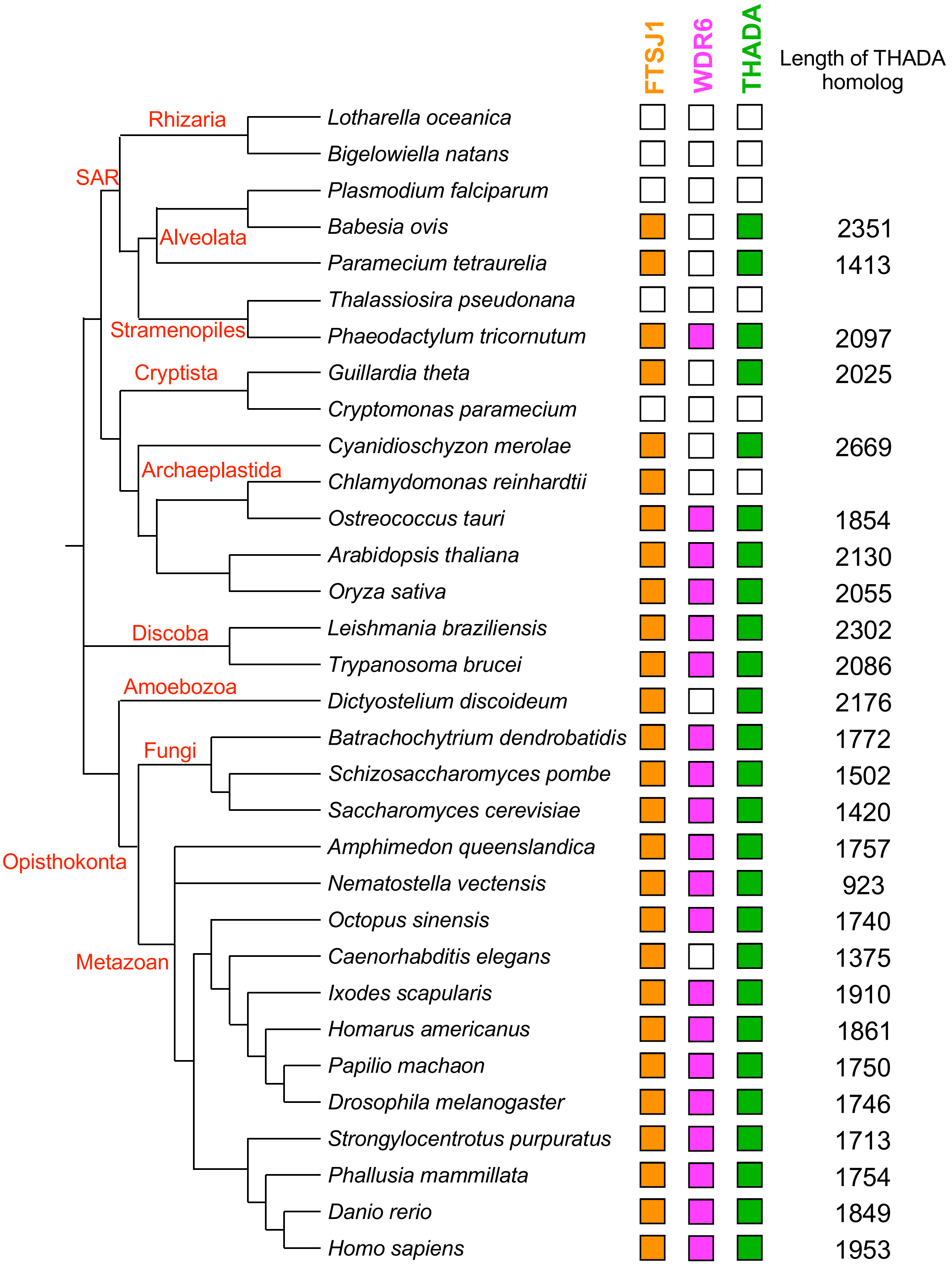

Supplementary Fig. 1 Phylogenetic distribution of FTSJ1, WDR6 and THADA. Phylogenetic tree showing evolutionary conservation of FTSJ1, WDR6, and THADA in representative eukaryotes. The presence and absence of the corresponding genes are indicated by the filled and open squares, respectively. The lengths of THADA homologs are also displayed.

(a)

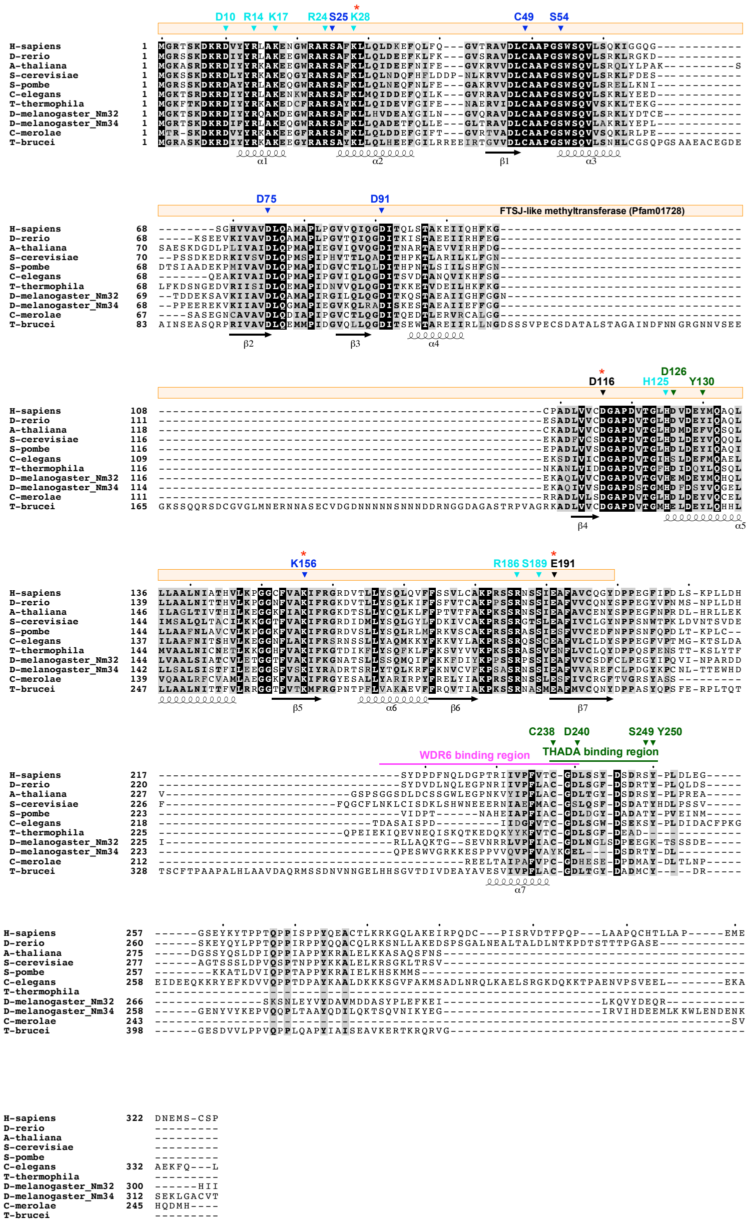
D10 R14 K17 R24 S25 K28 C49 S54
D75 D91
FTSJ-like methyltransferase (Pfam01728)
D116 H125 D126 Y130
K156 R186 S189 E191
C238 D240 S249 Y250
WDR6 binding region
THADA binding region
H-sapiens
D-rerio
A-thaliana
S-cerevisiae
S-pombe
C-elegans
T-thermophila
D-melanogaster_Nm32
D-melanogaster_Nm34
C-merolae
T-brucei

(b)

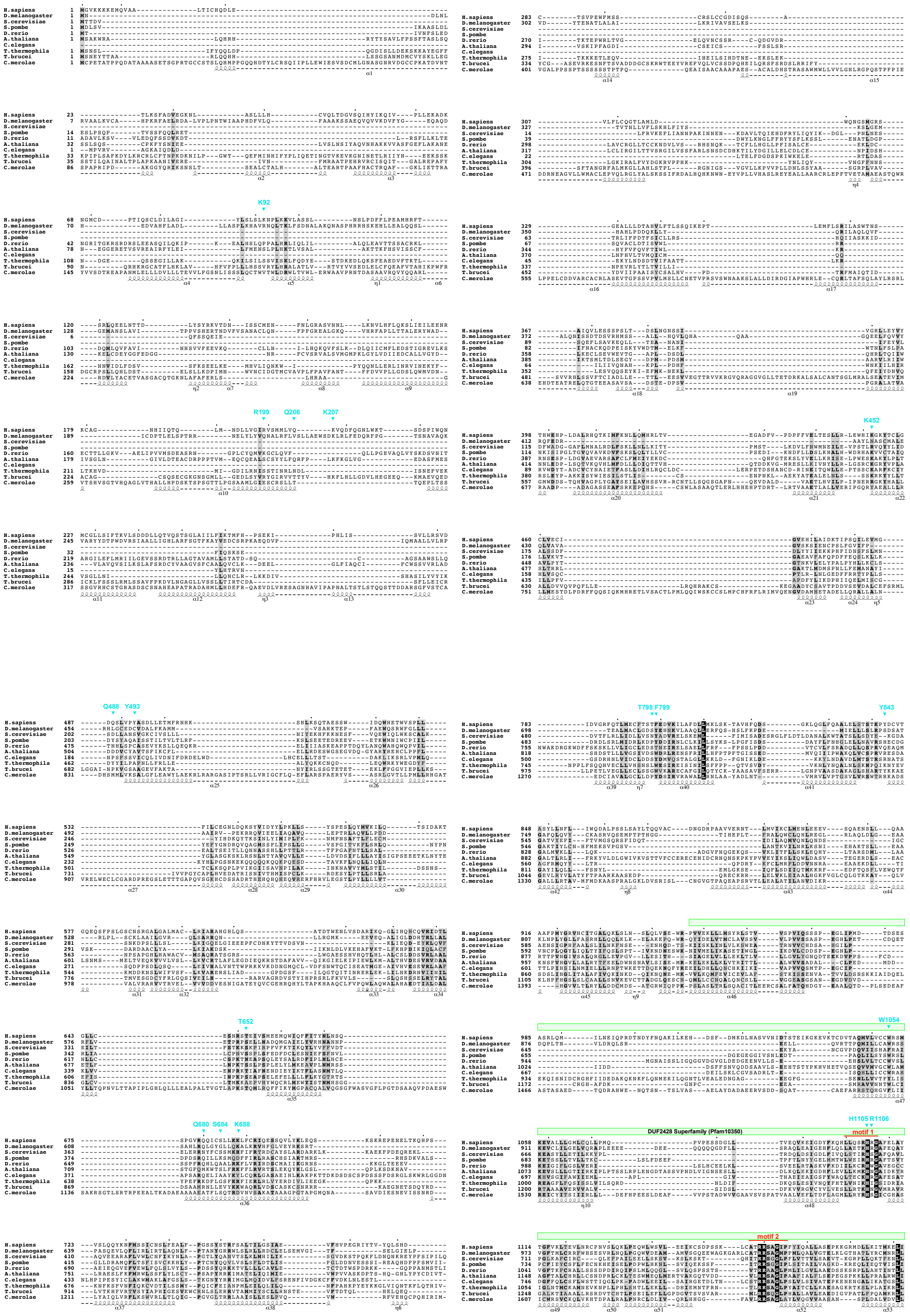

K92
R199
Q206
K207
K482
Q488
Y493
T798
F799
Y843
T652
W1054
Q680
S684
K688
H1105
R1106
DUF2428 Superfamily (Pfam10350)
motif 1
motif 2

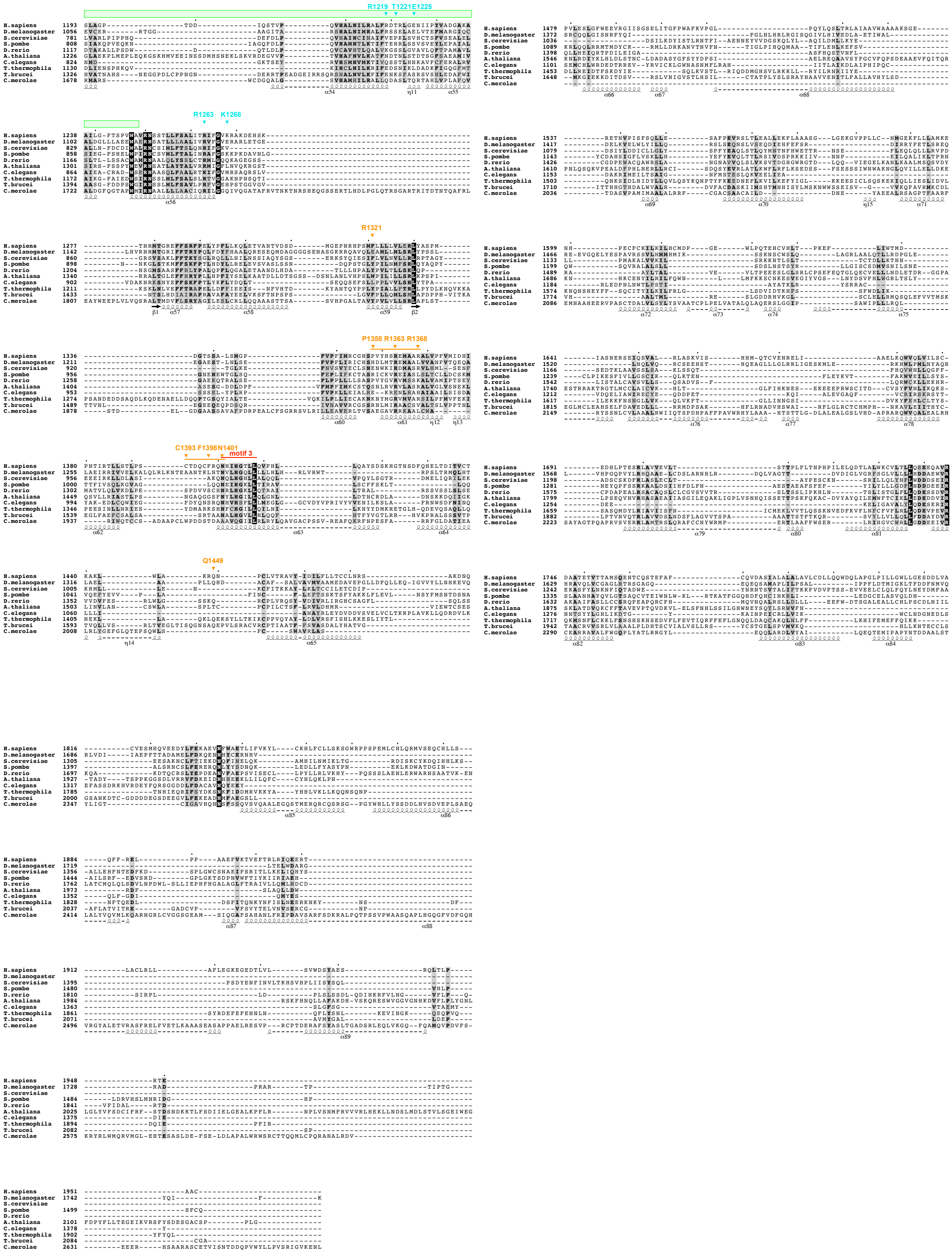

Supplementary Fig. 2 Sequence alignment of FTSJ1 or THADA homologs.

Multiple alignments of 11 FTSJ1 orthologs **(a)** and 10 THADA orthologs **(b)** using MAFFT[1] and EsPript (https://espript.ibcp.fr)[2]. Conserved domains are indicated by a box above the alignment. The secondary structures defined by our model are as follows. Binding regions for FTSJ1, THADA, WDR6, tRNA, and SAH are indicated in orange, green, pink, cyan, and blue, respectively. Residues previously reported as catalytic sites for FTSJ1[3] are marked with asterisks.

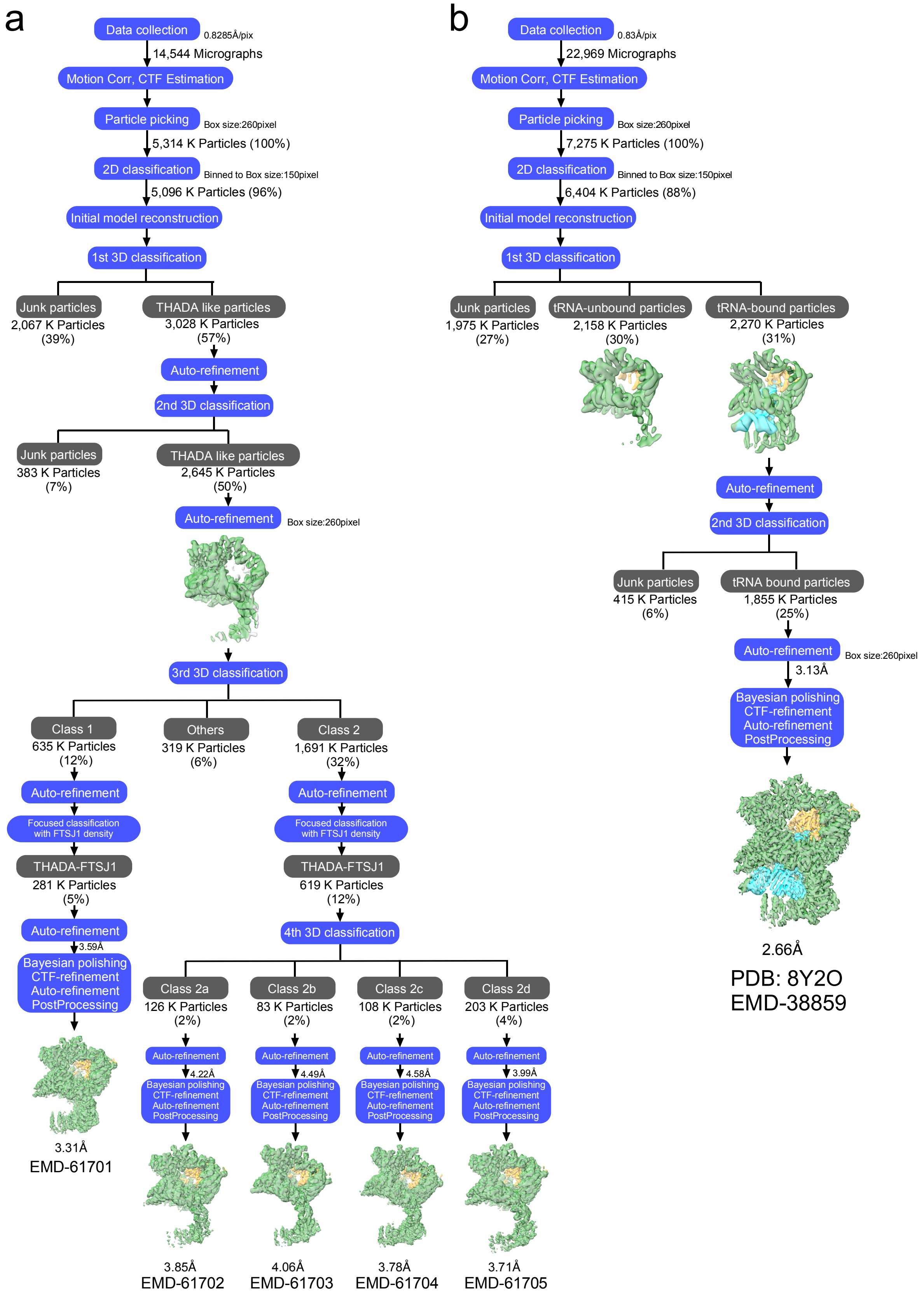

Supplementary Fig. 3 Image processing procedures of cryo-EM analysis

Image processing procedures of FTSJ1-THADA **(a)** and FTSJ1-THADA-tRNA^{Phe} complex **(b)**.

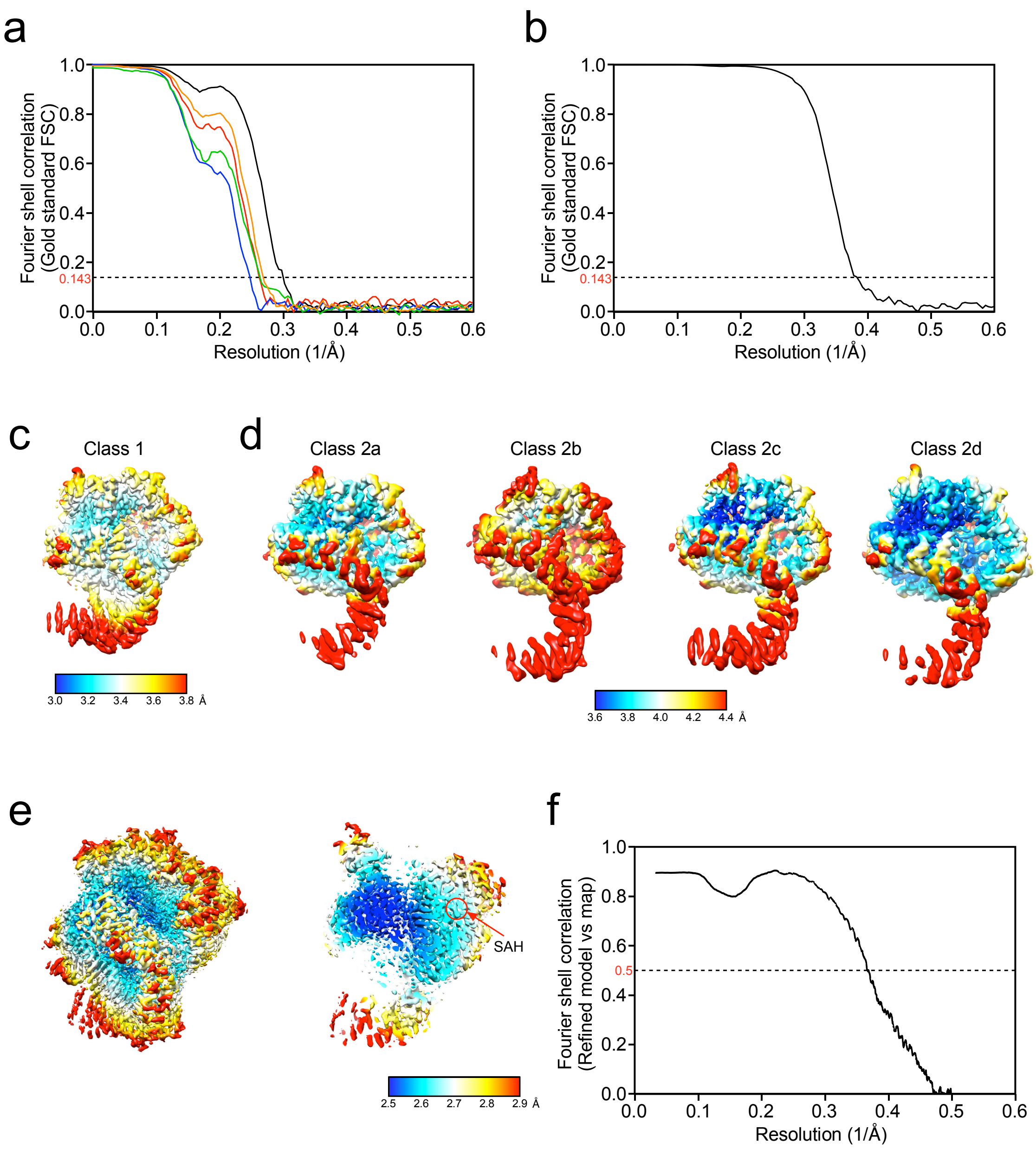

Supplementary Fig. 4 Local resolution and FSC curve of cryo-EM analysis

a, Gold standard Fourier shell correlation (FSC) curves of the maps of FTSJ1-THADA. Black, Class 1 (EMDB: EMD-61701); red, Class 2a (EMDB: EMD-61702); blue, Class 2b (EMDB: EMD-61703); green, Class 2c (EMDB: EMD-61704); orange, Class 2d (EMDB: EMD-61705).

b, Gold standard FSC curve of FTSJ1-THADA-tRNA^{Phe} complex (PDB:8Y2O, EMD-38859).

c,d, Color-coded local resolution distribution of the indicated cryo-EM maps of FTSJ1-THADA.

e, Color-coded local resolution distribution of the cryo-EM maps of the FTSJ1-THADA-tRNA^{Phe} complex (PDB:8Y2O, EMD-38859). The right panel shows the cross-sectional resolution, including the SAH binding site.

f, Refined model versus map of the FSC curve of the FTSJ1-THADA-tRNA^{Phe} complex (PDB:8Y2O, EMD-38859).

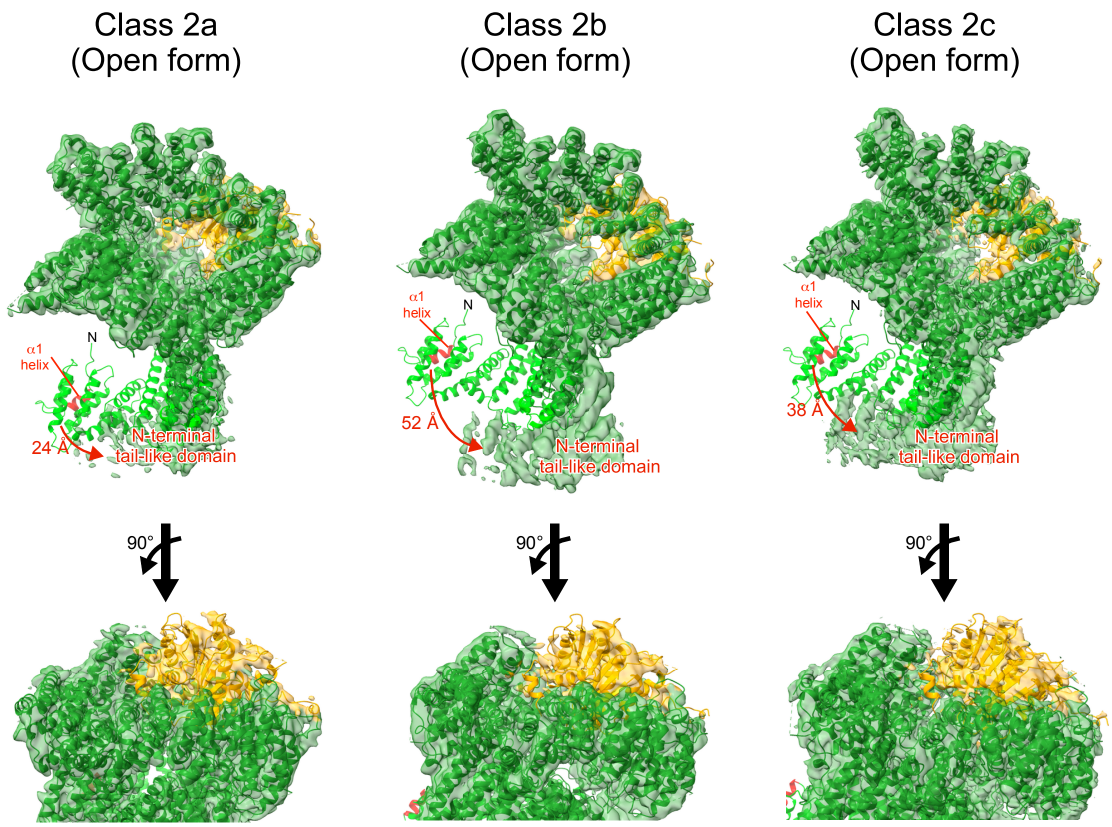

Supplementary Fig. 5 Cryo-EM maps of FTSJ1-THADA in open form

Each map is superimposed on the closed-form model of FTSJ1-THADA. The bottom images show a close-up view of the top of THADA. FTSJ1 is indicated in orange and THADA in green. The N-terminal tail-like domain of THADA (1-344) is in light green and α1 helix of THADA (18–25) is in red.

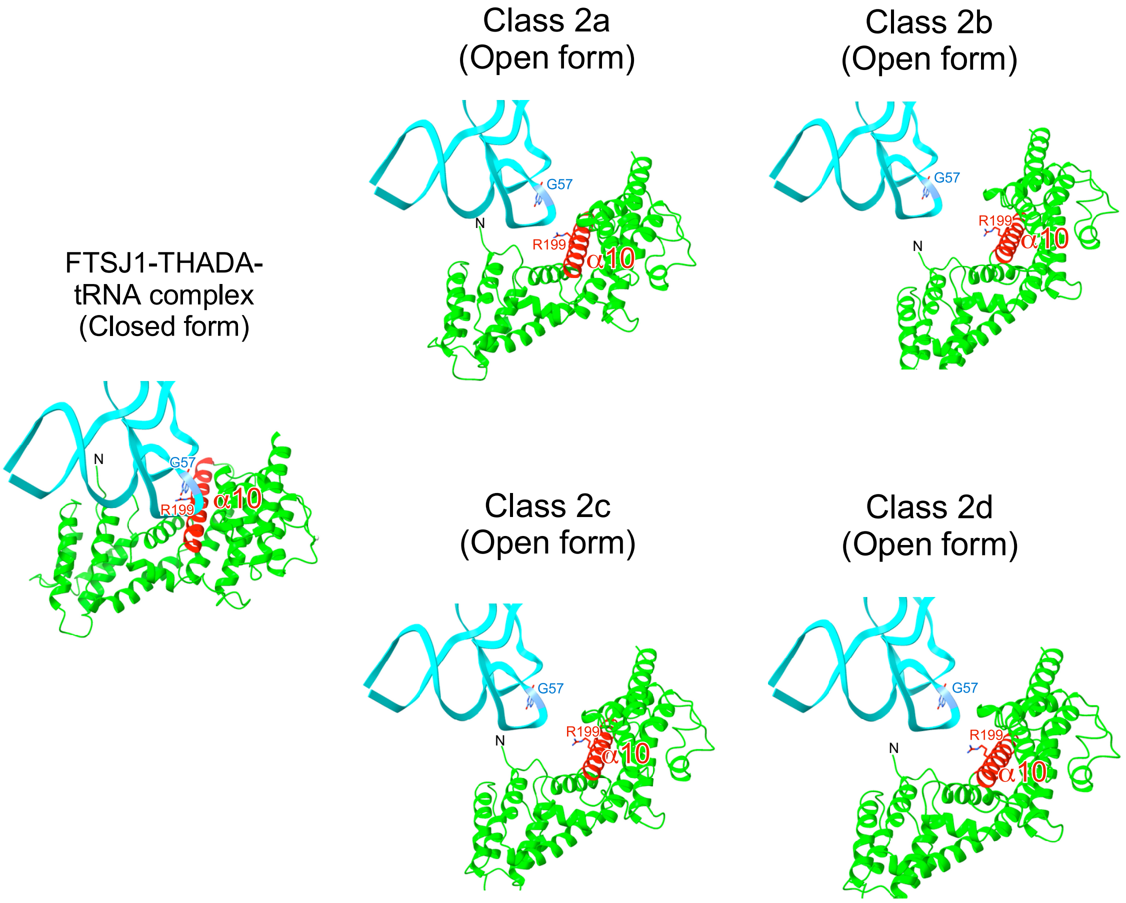

Supplementary Fig. 6 The relative position of the N-terminal tail-like domain to the tRNA substrate

Comparing the relative position of the N-terminal tail-like domain of THADA (1–344) to the tRNA substrate among five structures. Left: the contact site between tRNA and the N-terminal tail-like domain of THADA (PDB:8Y2O). Right: each indicated map is superimposed on the closed-form model of FTSJ1-THADA. The position of the N-terminal tail-like domain of THADA (1–344) of the indicated map is shown. THADA and tRNA are colored in light green and cyan, respectively. α10 helix of THADA (184–206) is highlighted in red. G57 of tRNA and Arg199 of THADA are represented as stick models.

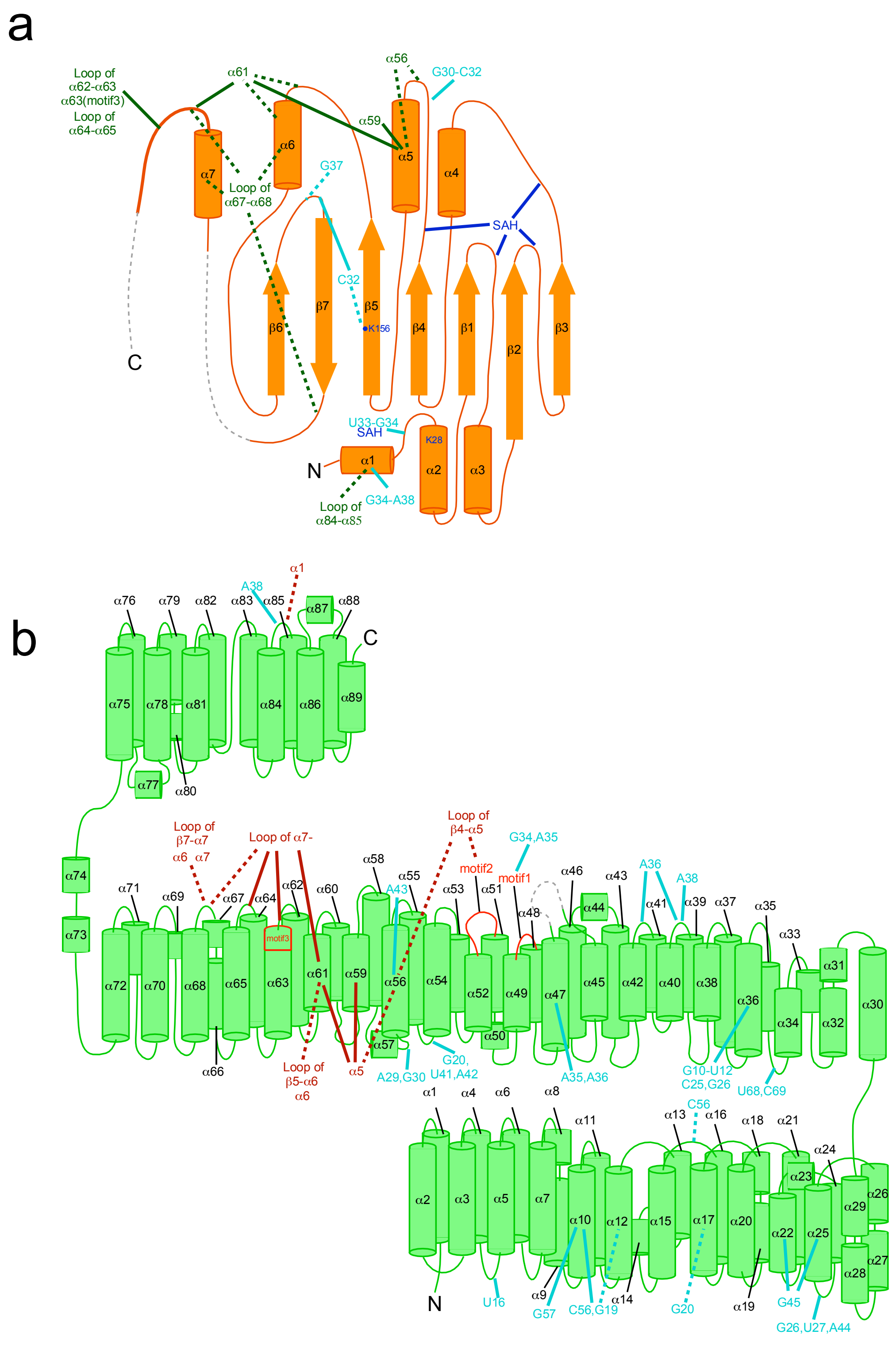

Supplementary Fig. 7 Topological diagram of FTSJ1 (a) and THADA (b)

The α-helices and β-strands are shown. The N- and C- terminal ends are denoted as N and C, respectively. The conserved motifs of THADA[4] are indicated in red. The contact regions of FTSJ1, THADA, tRNA, and SAH are indicated in orange, green, cyan, and blue, respectively. Hydrogen bonds are depicted as solid lines, and hydrophobic interactions are depicted as dotted lines.

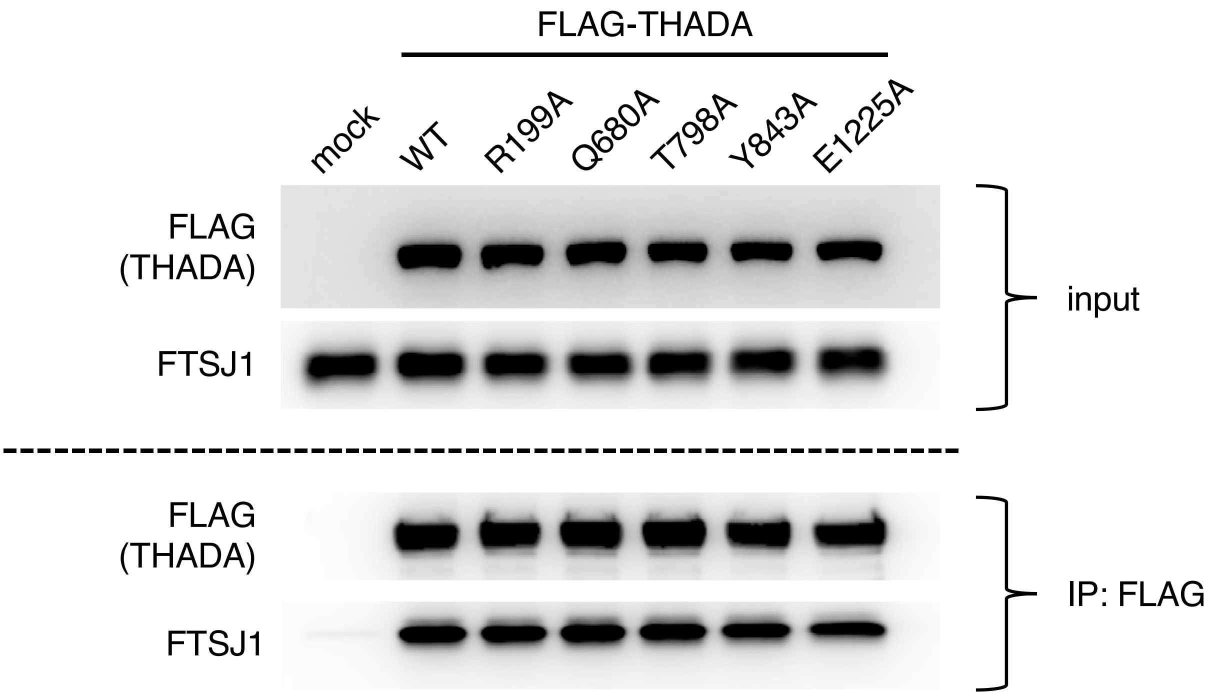

Supplementary Fig. 8 Evaluation of the complex formation between FTSJ1 and THADA mutants

Immunoprecipitation was performed using the anti-FLAG antibody in 293FT cells overexpressing FLAG-tagged wild-type THADA or mutant THADA. Immunoprecipitants were subjected to immunoblotting with anti-FLAG or anti-FTSJ1 antibodies. THADA is biochemically bound to endogenous FTSJ1. Unedited blot image is also provided as Supplementary Fig. 12.

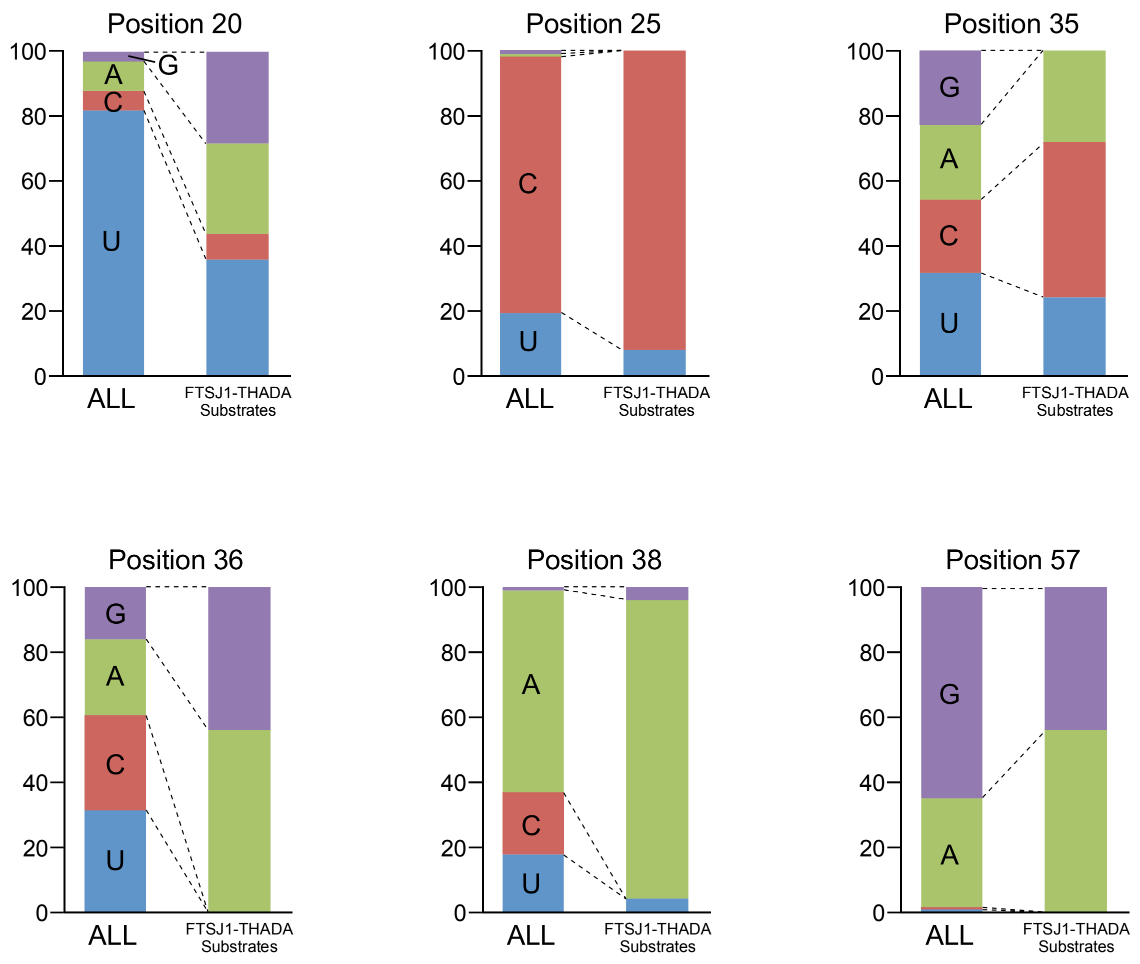

Supplementary Fig. 9 Sequence biases at FTSJ1-THADA recognizing positions

Uridine, cytidine, adenosine, and guanosine are shown in blue, red, green, and purple, respectively. tRNA sequences were obtained from GtRNAdb[5] and manually aligned. Pseudogenes and duplicate genes were excluded.

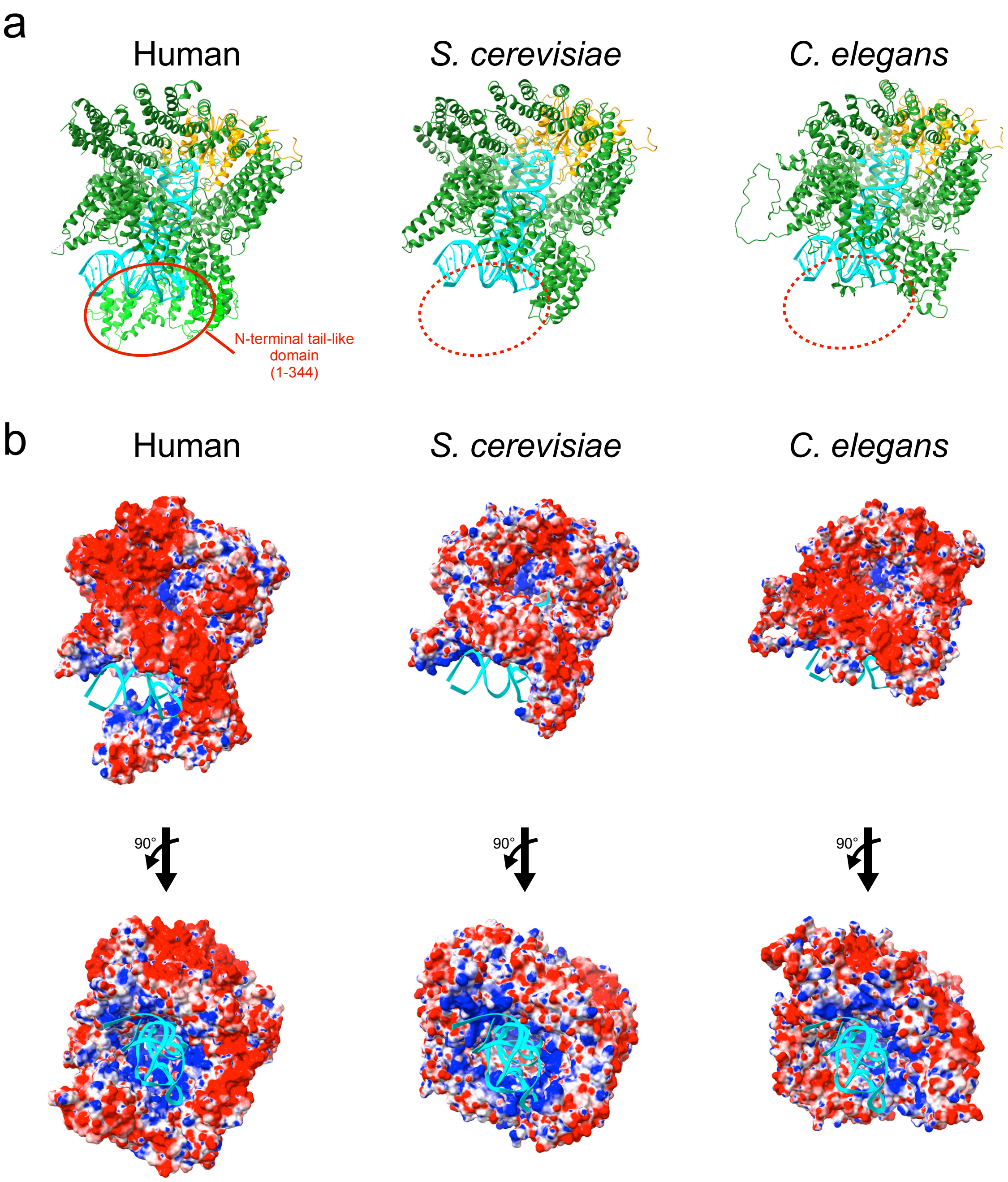

Supplementary Fig. 10 Comparison among THADA homologs

a, Comparison of overall structure of human THADA with the AlphaFold model of the THADA homolog in *S. cerevisiae* (Q03496) or *C. elegans* (W6RTG0)[6,7] superimposed with the human FTSJ1-THADA-tRNA^{Phe} complex (PDB:8Y2O). FTSJ1, THADA, and tRNA are depicted in orange, green and cyan, respectively. The N-terminal tail-like domain of THADA (1–344) is highlighted in light green.

b, Electrostatic surface potential of THADA homologs generated by the Adaptive Poisson-Boltzmann Solver (APBS) software[8] overlaid on the tRNA substrate model (cyan), viewed from the side (upper) or below (lower). Positively and negatively charged areas are indicated blue and red, respectively.

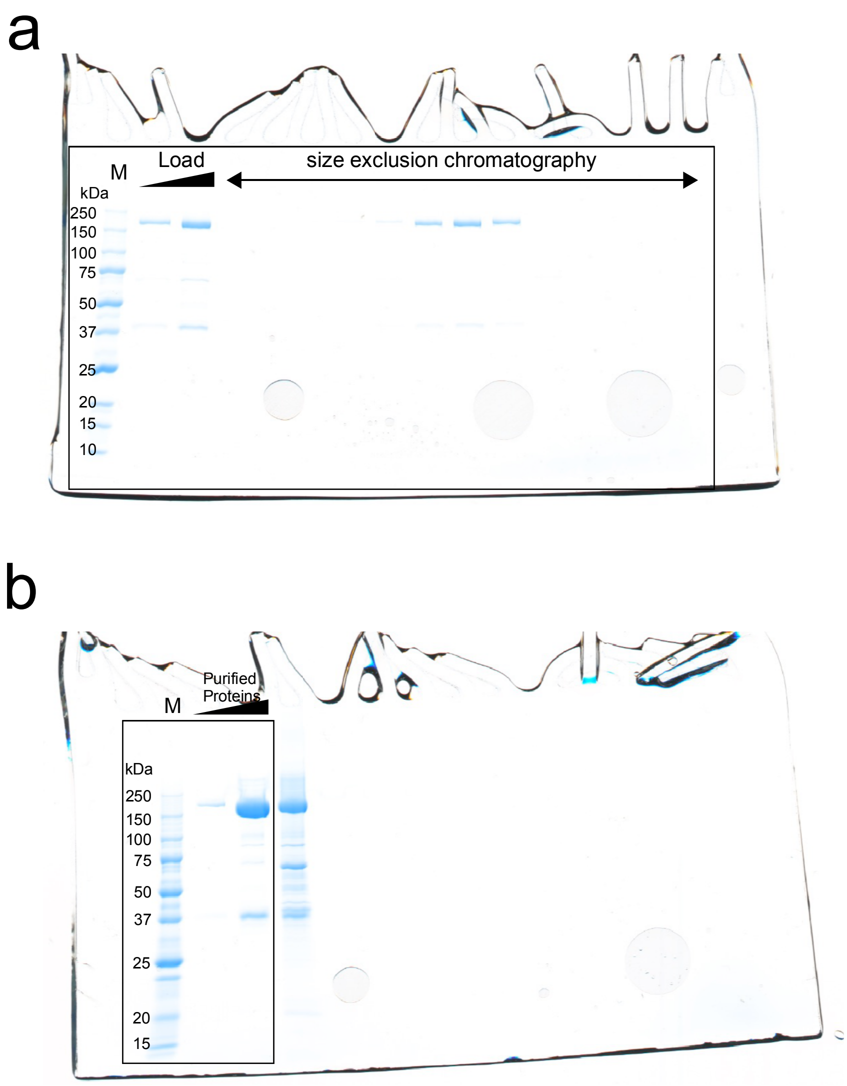

Supplementary Fig. 11 Unedited image of SDS-PAGE gel in Figure 1b.

Unedited image of left gel **(a)** or right gel **(b)** in Fig. 1b. The boxed area is the area shown in Figure 1b.

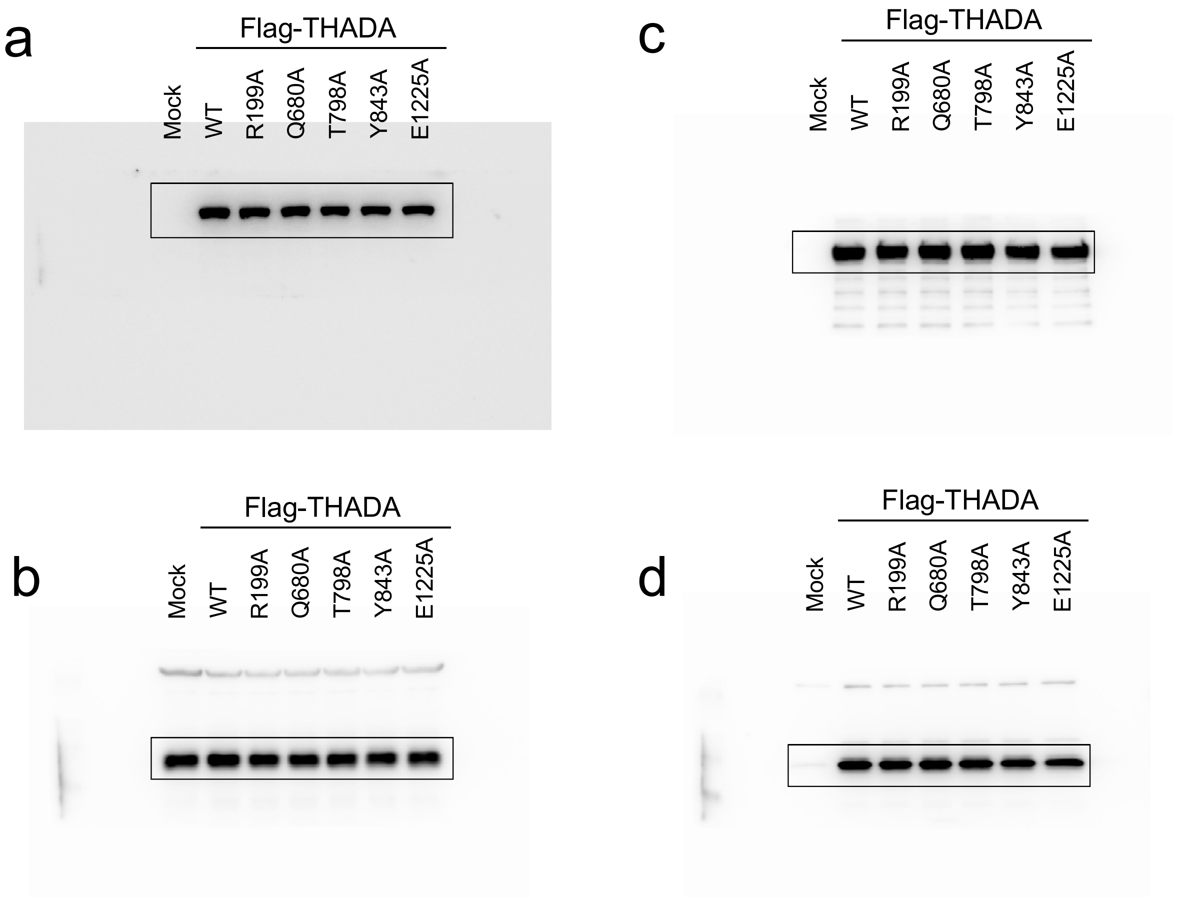

Supplementary Fig. 12 Unedited image of immunoblotting in Supplementary Figure 8.

Unedited image of input **(a,b)** or IP **(c,d)** blot in Supplementary Fig. 8. Immunoprecipitants were subjected to immunoblotting with anti-FLAG **(a,c)** or anti-FTSJ1 **(b,d)** antibodies. The boxed area is the area shown in Supplementary Fig. 8.

Table S1. Nm32 and Nm34 detection in human tRNAs

a,

tRNA Species	M. Brazane (2023)	Y. Nagayoshi (2021)	J. Li (2020)	MP. Guy (2015)
**Arg (UCG1)**	**Cm**	no data	**Cm**	no data
**Arg (UCG2)**	**Cm**	no data	no data	no data
**Arg (CCG)**	**Um**	no data	**Um/Cm**	no data
**Arg (ACG)**	**Cm**	no data	**Cm**	no data
**Leu (CAG)**	**Um**	no data	no data	no data
**Leu (AAG)**	**Um**	no data	no data	no data
**Leu (UAG)**	**Um**	no data	no data	no data
**Phe (GAA)**	**Cm**	**Cm**	**Cm**	**Cm**
**Trp (CCA)**	**Cm**	**Cm**	**Cm**	**Cm**
**Gln (CUG)**	**Cm**	no data	no data	no data
**Gln (UUG)**	**Cm**	no data	**Cm**	no data
**Gly (CCC)**	**Um**	no data	not detected	no data
**Cys (GCA)**	**Cm**	no data	no data	no data
**Pro (UGG)**	**Um**	no data	no data	no data

b,

tRNA Species	M. Brazane (2023)	Y. Nagayoshi (2021)	J. Li (2020)	L. Kawarada (2017)	MP. Guy (2015)
**Phe (GAA)**	**Gm**	**Gm**	**Gm**	no data	**Gm**
**Leu (CAA)**	not detected	no data	no data	**Cm**	no data
**Leu (UAA)**	**Um**	no data	not detected	no data	no data
**Trp (CCA)**	**Cm**	**Cm**	**Cm (not depend on wdr6)**	no data	**Cm**
**Sec (UCA)**	no data	**Um**	no data	no data	no data

List of human tRNA species in which Nm32(a) and Nm34(b) are detected in previous studies[9-13]. The tRNA species detected in two or more studies are highlighted in red.

Table S2. List of synthetic oligonucleotides used in this study

Purpose	Construct	Sequence (5' to 3')
**PCR amplification of T7 template**	Phe_Fw	GCTAATACGACTCACTATAGGGCCGAAATAGCTCAGTTGGGAGAG
	Phe_Body	AATAGCTCAGTTGGGAGAGCGTTAGACTGAAGATCTAAAGGTCCC
	Phe_Rv	TGGTGCCGAAACCCGGGATCGAACCAGGGACCTTTAGATCTTCAG
**Plasmid construction**	THADA_Fw	CAATTTGAGAAAGGCGCTGAAAACCTGTATTTTCAGGGCATGGGTGTAAAGAAGAAGAAAGAAATGC
	THADA_Rv	AGGGATCGAACCCTTCATTAACATGCCGCTTCTGTTCTTGGAAG
	FTSJ1_Fw	AAAACCTGTATTTTCAGGGCATGGGTCGAACTAGTAAGGATAAGCGAGATGTCTATTATAGACTTGC
	FTSJ1_Rv	AGGGATCGAACCCTTCATTATGGACTGCATGACATCTCATTGTCCTCCATTTCAG
**THADA mutant construction**	R199A_Fw	GACTTACTGGTAGGCATTGCAGTTTCAATG
	R199A_Rv	CTTTCTGTACTAACATCATTGAAACTGCAATGC
	Q680A_Fw	CCAGTCTCCAGGAGTGCGGGCACAGATC
	Q680A_Rv	CTTTTTAAGAAGAGAACAGATCTGTGCCCGCAC
	T798A_Fw	GGAATGTTTTACCAGCGCTTTTGAAGAC
	T798A_Rv	GCTAAAATTTTCACGTCTTCAAAAGCGCTG
	Y843A_Fw	CTCAGCACAAGCACCAAACCAGCCGACTG
	Y843A_Rv	GGAAGCTGTCACACAGTCGGCTGGTTTG
	E1225A_Fw	CAGAGATACGCGCCTGGGAGCAAATATTATTC
	E1225A_Rv	CAGCAACATAAGGAATAATATTTGCTCCCAG

Supplementary References

1 Katoh, K., Rozewicki, J. & Yamada, K. D. MAFFT online service: multiple sequence alignment, interactive sequence choice and visualization. *Brief Bioinform* **20**, 1160-1166 (2019). https://doi.org:10.1093/bib/bbx108

2 Robert, X. & Gouet, P. Deciphering key features in protein structures with the new ENDscript server. *Nucleic Acids Res* **42**, W320-324 (2014). https://doi.org:10.1093/nar/gku316

3 Hager, J., Staker, B. L., Bugl, H. & Jakob, U. Active site in RrmJ, a heat shock-induced methyltransferase. *J Biol Chem* **277**, 41978-41986 (2002). https://doi.org:10.1074/jbc.M205423200

4 Funk, H. M. *et al.* Identification of a Trm732 Motif Required for 2'. *ACS Omega* **7**, 13667-13675 (2022). https://doi.org:10.1021/acsomega.1c07231

5 Thornlow, B. P. *et al.* Predicting transfer RNA gene activity from sequence and genome context. *Genome Res* **30**, 85-94 (2020). https://doi.org:10.1101/gr.256164.119

6 Jumper, J. *et al.* Highly accurate protein structure prediction with AlphaFold. *Nature* **596**, 583-589 (2021). https://doi.org:10.1038/s41586-021-03819-2

7 Varadi, M. *et al.* AlphaFold Protein Structure Database: massively expanding the structural coverage of protein-sequence space with high-accuracy models. *Nucleic Acids Res* **50**, D439-D444 (2022). https://doi.org:10.1093/nar/gkab1061

8 Jurrus, E. *et al.* Improvements to the APBS biomolecular solvation software suite. *Protein Sci* **27**, 112-128 (2018). https://doi.org:10.1002/pro.3280

9 Brazane, M. *et al.* The ribose methylation enzyme FTSJ1 has a conserved role in neuron morphology and learning performance. *Life Sci Alliance* **6** (2023). https://doi.org:10.26508/lsa.202201877

10 Nagayoshi, Y. *et al.* Loss of Ftsj1 perturbs codon-specific translation efficiency in the brain and is associated with X-linked intellectual disability. *Sci Adv* **7** (2021). https://doi.org:10.1126/sciadv.abf3072

11 Li, J. *et al.* Intellectual disability-associated gene ftsj1 is responsible for 2'-O-methylation of specific tRNAs. *EMBO Rep* **21**, e50095 (2020). https://doi.org:10.15252/embr.202050095

12 Guy, M. P. *et al.* Defects in tRNA Anticodon Loop 2'-O-Methylation Are Implicated in Nonsyndromic X-Linked Intellectual Disability due to Mutations in FTSJ1. *Hum Mutat* **36**, 1176-1187 (2015). https://doi.org:10.1002/humu.22897

13 Kawarada, L., Suzuki, T., Ohira, T., Hirata, S. & Miyauchi, K. ALKBH1 is an RNA dioxygenase responsible for cytoplasmic and mitochondrial tRNA modifications. *Nucleic Acids Res* **45**, 7401-7415 (2017). https://doi.org:10.1093/nar/gkx354
