# Supplementary material for: Structural insights into tRNA recognition of the human FTSJ1-THADA complex
Source: Commun Biol. 2025 Jun 7;8:893. doi: 10.1038/s42003-025-08278-3 (PMC12145424; doi:10.1038/s42003-025-08278-3)
Supplement: Supplementary file 3 — Description of Additional Supplementary Files [file 42003_2025_8278_MOESM3_ESM.pdf]

Description of Additional Supplementary Files

File name: Supplementary Data 1.

Description: Comparison of sequence biases between all tRNAs and FTSJ1-THADA Substrates

File name: Supplementary Data 2.

Description: The raw data of methyltransferase assay of FTSJ1-THADA in Figure 5e.

File name: Supplementary Movie 1.

Description:3D variability analysis using all THADA-like particles (2645K particles) from the dataset of FTSJ1-THADA without the tRNA substrate.

File name: Supplementary Movie 2.

Description:3D variability analysis using Class 1 particles (635K particles) from the dataset of FTSJ1-THADA without the tRNA substrate.

File name: Supplementary Movie 3.

Description:3D variability analysis using Class 2 particles (1691K particles) from the dataset of FTSJ1-THADA without the tRNA substrate.

File name: Supplementary Movie 4.

Description:3D variability analysis using tRNAbound FTSJ1-THADA particles (2270K particles) from the dataset of FTSJ1-THADA with the tRNA substrate.

File name: 8Y2O_EMD-38859_FTSJ1-THADA_tRNA.pdf

Description: Validation report for the structure of FTSJ1-THADA with tRNA (PDB:8Y2O, EMD-38859).

File name: EMD-61701_FTSJ1-THADA_Class1.pdf

Description: Validation report for the structure of FTSJ1-THADA Class 1 (EMD-61701).

File name: EMD-61702_FTSJ1-THADA_Class2a.pdf

Description: Validation report for the structure of FTSJ1-THADA Class 2a (EMD-61702).

File name: EMD-61703_FTSJ1-THADA_Class2b.pdf

Description: Validation report for the structure of FTSJ1-THADA Class 2b (EMD-61703).

File name: EMD-61704_FTSJ1-THADA_Class2c.pdf

Description: Validation report for the structure of FTSJ1-THADA Class 2c (EMD-61704).

File name: EMD-61705_FTSJ1-THADA_Class2d.pdf

Description: Validation report for the structure of FTSJ1-THADA Class 2d (EMD-61705).
