# Supplementary material for: Structural insights into tRNA recognition of the human FTSJ1-THADA complex
Source: Commun Biol. 2025 Jun 7;8:893. doi: 10.1038/s42003-025-08278-3 (PMC12145424; doi:10.1038/s42003-025-08278-3)
Supplement: Supplementary file 10 — 8Y2O_EMD-38859_FTSJ1-THADA_tRNA [file 42003_2025_8278_MOESM10_ESM.pdf]

Full wwPDB EM Validation Report (i)

Aug 28, 2024 – 02:36 PM JST

PDB ID	:	8Y2O
EMDB ID	:	EMD-38859
Title	:	The Cryo-EM structure of human tRNA methyltransferase FTSJ1-THADA with substrate tRNA and S-adenosyl homocysteine (SAH)
Deposited on	:	2024-01-26
Resolution	:	2.66 Å(reported)

This wwPDB validation report is for manuscript review

This is a Full wwPDB EM Validation Report.

This report is produced by the wwPDB biocuration pipeline after annotation of the structure.

We welcome your comments at *validation@mail.wwpdb.org*
A user guide is available at
https://www.wwpdb.org/validation/2017/EMValidationReportHelp
with specific help available everywhere you see the (i) symbol.

The types of validation reports are described at
http://www.wwpdb.org/validation/2017/FAQs#types.

The following versions of software and data (see references (i)) were used in the production of this report:

EMDB validation analysis	:	0.0.1.dev112
Mogul	:	1.8.5 (274361), CSD as541be (2020)
MolProbity	:	4.02b-467
buster-report	:	1.1.7 (2018)
Percentile statistics	:	20231227.v01 (using entries in the PDB archive December 27th 2023)
MapQ	:	1.9.13
Ideal geometry (proteins)	:	Engh & Huber (2001)
Ideal geometry (DNA, RNA)	:	Parkinson et al. (1996)

For Manuscript Review

1 Overall quality at a glance ⓘ

The following experimental techniques were used to determine the structure:
ELECTRON MICROSCOPY

The reported resolution of this entry is 2.66 Å.

Percentile scores (ranging between 0-100) for global validation metrics of the entry are shown in the following graphic. The table shows the number of entries on which the scores are based.

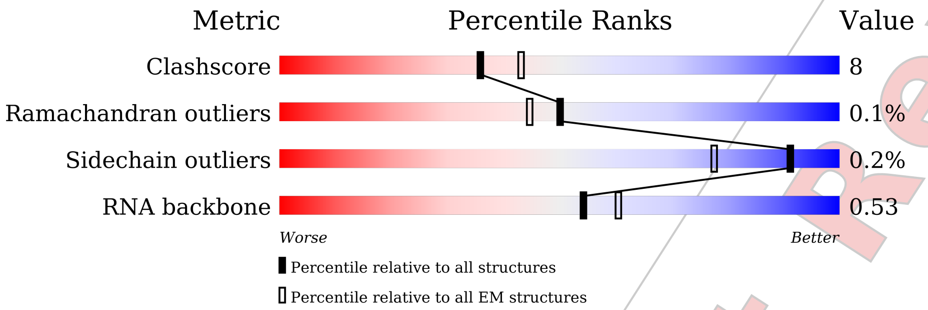

Metric	Whole archive (#Entries)	EM structures (#Entries)
Clashscore	210492	15764
Ramachandran outliers	207382	16835
Sidechain outliers	206894	16415
RNA backbone	6643	2191

The table below summarises the geometric issues observed across the polymeric chains and their fit to the map. The red, orange, yellow and green segments of the bar indicate the fraction of residues that contain outliers for >=3, 2, 1 and 0 types of geometric quality criteria respectively. A grey segment represents the fraction of residues that are not modelled. The numeric value for each fraction is indicated below the corresponding segment, with a dot representing fractions <=5% The upper red bar (where present) indicates the fraction of residues that have poor fit to the EM map (all-atom inclusion < 40%). The numeric value is given above the bar.

Mol	Chain	Length	Quality of chain
1	A	1981	8% / 75% 15% 10%
2	B	347	• / 46% 18% 35%
3	C	76	• / 67% 20% 8% 5%

For Manuscript Review

2 Entry composition (i)

There are 6 unique types of molecules in this entry. The entry contains 17385 atoms, of which 0 are hydrogens and 0 are deuteriums.

In the tables below, the AltConf column contains the number of residues with at least one atom in alternate conformation and the Trace column contains the number of residues modelled with at most 2 atoms.

- Molecule 1 is a protein called tRNA (32-2'-O)-methyltransferase regulator THADA.

Mol	Chain	Residues	Atoms					AltConf	Trace
			Total	C	N	O	S		
1	A	1786	14101	9025	2369	2604	103	1	0

There are 28 discrepancies between the modelled and reference sequences:

Chain	Residue	Modelled	Actual	Comment	Reference
A	-27	MET	-	initiating methionine	UNP Q6YHU6
A	-26	ASP	-	expression tag	UNP Q6YHU6
A	-25	TYR	-	expression tag	UNP Q6YHU6
A	-24	LYS	-	expression tag	UNP Q6YHU6
A	-23	ASP	-	expression tag	UNP Q6YHU6
A	-22	ASP	-	expression tag	UNP Q6YHU6
A	-21	ASP	-	expression tag	UNP Q6YHU6
A	-20	ASP	-	expression tag	UNP Q6YHU6
A	-19	LYS	-	expression tag	UNP Q6YHU6
A	-18	SER	-	expression tag	UNP Q6YHU6
A	-17	ALA	-	expression tag	UNP Q6YHU6
A	-16	TRP	-	expression tag	UNP Q6YHU6
A	-15	SER	-	expression tag	UNP Q6YHU6
A	-14	HIS	-	expression tag	UNP Q6YHU6
A	-13	PRO	-	expression tag	UNP Q6YHU6
A	-12	GLN	-	expression tag	UNP Q6YHU6
A	-11	PHE	-	expression tag	UNP Q6YHU6
A	-10	GLU	-	expression tag	UNP Q6YHU6
A	-9	LYS	-	expression tag	UNP Q6YHU6
A	-8	GLY	-	expression tag	UNP Q6YHU6
A	-7	ALA	-	expression tag	UNP Q6YHU6
A	-6	GLU	-	expression tag	UNP Q6YHU6
A	-5	ASN	-	expression tag	UNP Q6YHU6
A	-4	LEU	-	expression tag	UNP Q6YHU6
A	-3	TYR	-	expression tag	UNP Q6YHU6
A	-2	PHE	-	expression tag	UNP Q6YHU6
A	-1	GLN	-	expression tag	UNP Q6YHU6
A	0	GLY	-	expression tag	UNP Q6YHU6

For Manuscript Review

- Molecule 2 is a protein called tRNA (cytidine(32)/guanosine(34)-2'-O)-methyltransferase.

Mol	Chain	Residues	Atoms					AltConf	Trace
			Total	C	N	O	S		
2	B	224	1708	1090	292	317	9	0	0

There are 18 discrepancies between the modelled and reference sequences:

Chain	Residue	Modelled	Actual	Comment	Reference
B	-17	MET	-	initiating methionine	UNP Q9UET6
B	-16	ASP	-	expression tag	UNP Q9UET6
B	-15	TYR	-	expression tag	UNP Q9UET6
B	-14	LYS	-	expression tag	UNP Q9UET6
B	-13	ASP	-	expression tag	UNP Q9UET6
B	-12	ASP	-	expression tag	UNP Q9UET6
B	-11	ASP	-	expression tag	UNP Q9UET6
B	-10	ASP	-	expression tag	UNP Q9UET6
B	-9	LYS	-	expression tag	UNP Q9UET6
B	-8	GLY	-	expression tag	UNP Q9UET6
B	-7	ALA	-	expression tag	UNP Q9UET6
B	-6	GLU	-	expression tag	UNP Q9UET6
B	-5	ASN	-	expression tag	UNP Q9UET6
B	-4	LEU	-	expression tag	UNP Q9UET6
B	-3	TYR	-	expression tag	UNP Q9UET6
B	-2	PHE	-	expression tag	UNP Q9UET6
B	-1	GLN	-	expression tag	UNP Q9UET6
B	0	GLY	-	expression tag	UNP Q9UET6

- Molecule 3 is a RNA chain called human cytoplasmic tRNA(Phe).

Mol	Chain	Residues	Atoms					AltConf	Trace
			Total	C	N	O	P		
3	C	72	1542	687	277	506	72	0	0

There are 3 discrepancies between the modelled and reference sequences:

Chain	Residue	Modelled	Actual	Comment	Reference
C	74	C	-	insertion	GB 29540470
C	75	C	-	insertion	GB 29540470
C	76	A	-	insertion	GB 29540470

- Molecule 4 is ZINC ION (three-letter code: ZN) (formula: Zn).

For Manuscript Review

Mol	Chain	Residues	Atoms		AltConf
			Total	Zn	
4	A	1	1	1	0

- Molecule 5 is S-ADENOSYL-L-HOMOCYSTEINE (three-letter code: SAH) (formula: $C_{14}H_{20}N_6O_5S$) (labeled as "Ligand of Interest" by depositor).

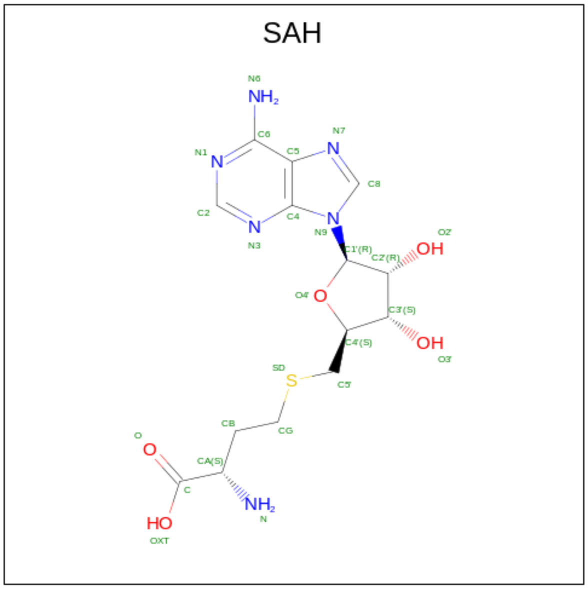

Mol	Chain	Residues	Atoms					AltConf
			Total	C	N	O	S	
5	B	1	26	14	6	5	1	0

- Molecule 6 is MAGNESIUM ION (three-letter code: MG) (formula: Mg).

Mol	Chain	Residues	Atoms		AltConf
			Total	Mg	
6	C	7	7	7	0

For Manuscript Review

3 Residue-property plots

These plots are drawn for all protein, RNA, DNA and oligosaccharide chains in the entry. The first graphic for a chain summarises the proportions of the various outlier classes displayed in the second graphic. The second graphic shows the sequence view annotated by issues in geometry and atom inclusion in map density. Residues are color-coded according to the number of geometric quality criteria for which they contain at least one outlier: green = 0, yellow = 1, orange = 2 and red = 3 or more. A red diamond above a residue indicates a poor fit to the EM map for this residue (all-atom inclusion < 40%). Stretches of 2 or more consecutive residues without any outlier are shown as a green connector. Residues present in the sample, but not in the model, are shown in grey.

• Molecule 1: tRNA (32-2'-O)-methyltransferase regulator THADA

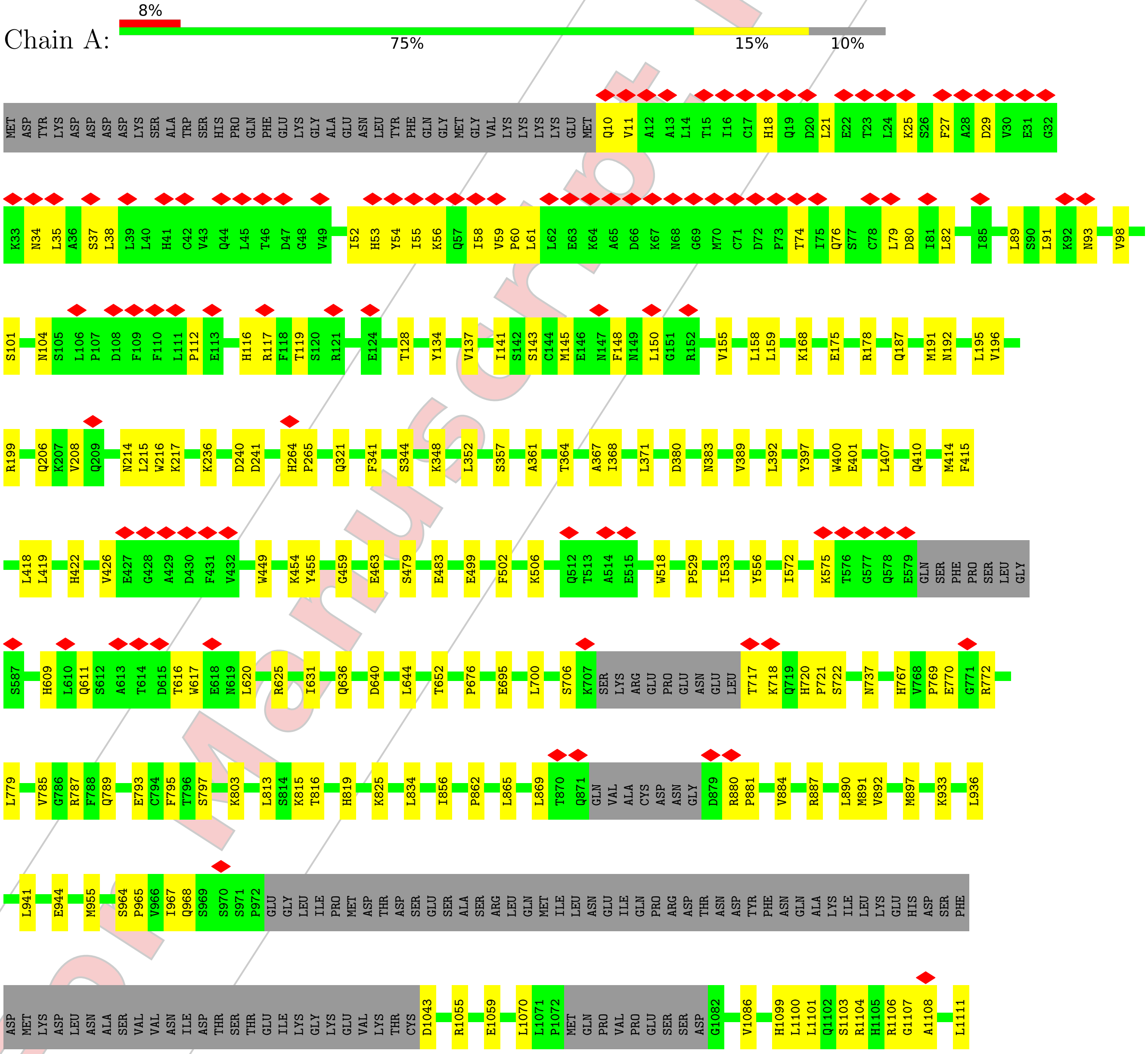

For Manuscript Review

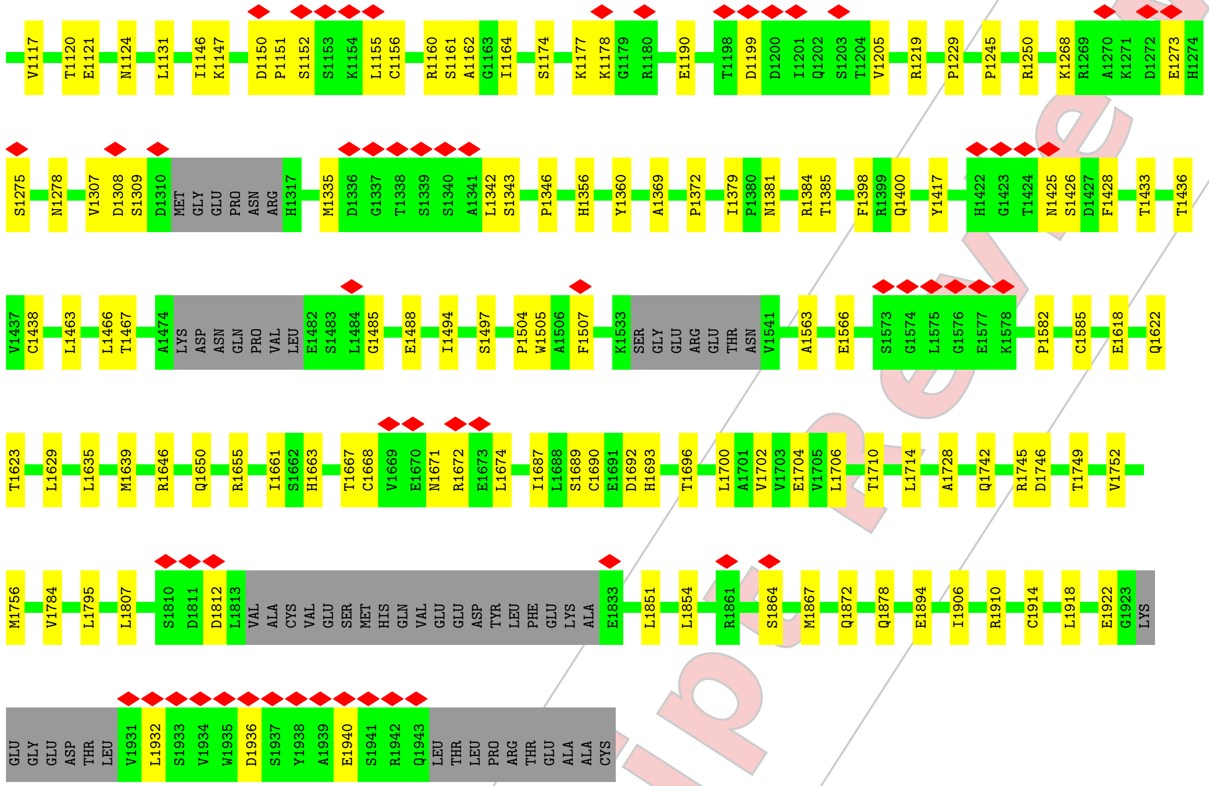

- Molecule 2: tRNA (cytidine(32)/guanosine(34)-2'-O)-methyltransferase

Chain B:
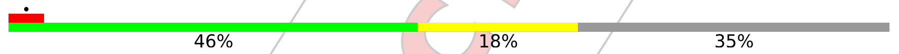

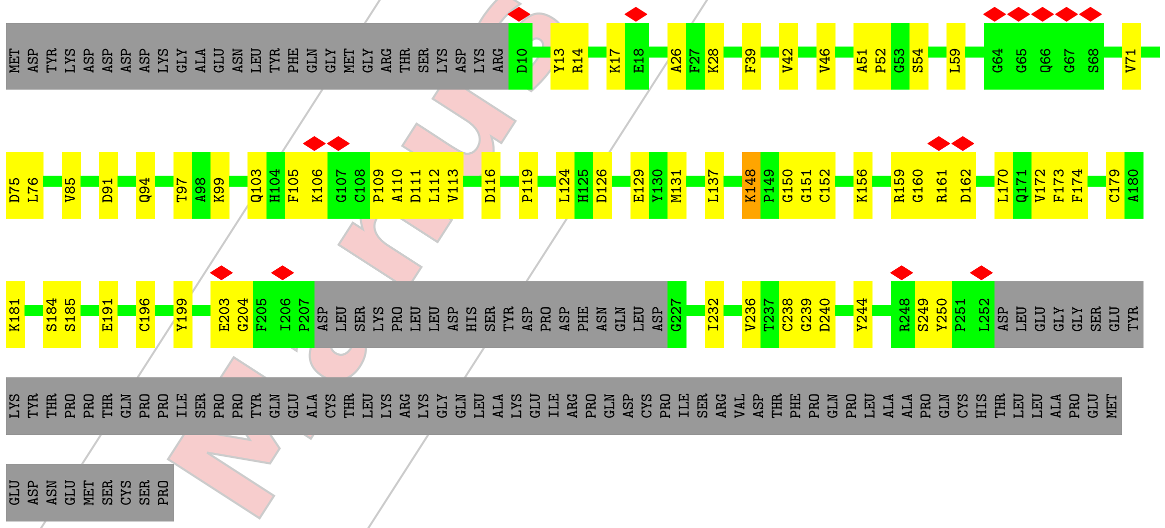

- Molecule 3: human cytoplasmic tRNA(Phe)

Chain C:
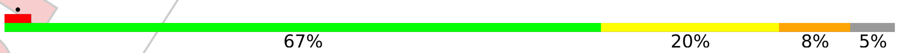

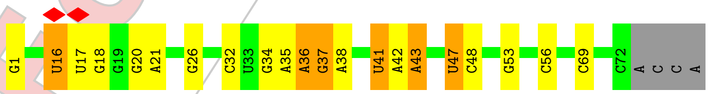

4 Experimental information (i)

Property	Value	Source
EM reconstruction method	SINGLE PARTICLE	Depositor
Imposed symmetry	POINT, C1	Depositor
Number of particles used	1854516	Depositor
Resolution determination method	FSC 0.143 CUT-OFF	Depositor
CTF correction method	PHASE FLIPPING AND AMPLITUDE CORRECTION	Depositor
Microscope	FEI TITAN KRIOS	Depositor
Voltage (kV)	300	Depositor
Electron dose ($e^-/\AA^2$)	50	Depositor
Minimum defocus (nm)	500	Depositor
Maximum defocus (nm)	2500	Depositor
Magnification	Not provided	
Image detector	GATAN K3 (6k x 4k)	Depositor
Maximum map value	0.106	Depositor
Minimum map value	-0.045	Depositor
Average map value	0.000	Depositor
Map value standard deviation	0.004	Depositor
Recommended contour level	0.0126	Depositor
Map size (Å)	215.8, 215.8, 215.8	wwPDB
Map dimensions	260, 260, 260	wwPDB
Map angles (°)	90.0, 90.0, 90.0	wwPDB
Pixel spacing (Å)	0.83, 0.83, 0.83	Depositor

For Manuscript Review

5 Model quality (i)

5.1 Standard geometry (i)

Bond lengths and bond angles in the following residue types are not validated in this section: SAH, MG, ZN

The Z score for a bond length (or angle) is the number of standard deviations the observed value is removed from the expected value. A bond length (or angle) with $|Z| > 5$ is considered an outlier worth inspection. RMSZ is the root-mean-square of all Z scores of the bond lengths (or angles).

Mol	Chain	Bond lengths		Bond angles	
		RMSZ	$\#\|Z\| > 5$	RMSZ	$\#\|Z\| > 5$
1	A	0.41	0/14391	0.47	0/19521
2	B	0.37	0/1745	0.47	0/2370
3	C	0.90	1/1724 (0.1%)	0.75	0/2685
All	All	0.48	1/17860 (0.0%)	0.51	0/24576

All (1) bond length outliers are listed below:

Mol	Chain	Res	Type	Atoms	Z	Observed(Å)	Ideal(Å)
3	C	1	G	OP3-P	-10.72	1.48	1.61

There are no bond angle outliers.

There are no chirality outliers.

There are no planarity outliers.

5.2 Too-close contacts (i)

In the following table, the Non-H and H(model) columns list the number of non-hydrogen atoms and hydrogen atoms in the chain respectively. The H(added) column lists the number of hydrogen atoms added and optimized by MolProbity. The Clashes column lists the number of clashes within the asymmetric unit, whereas Symm-Clashes lists symmetry-related clashes.

Mol	Chain	Non-H	H(model)	H(added)	Clashes	Symm-Clashes
1	A	14101	0	14277	208	0
2	B	1708	0	1702	48	0
3	C	1542	0	776	15	0
4	A	1	0	0	0	0
5	B	26	0	18	4	0
6	C	7	0	0	0	0
All	All	17385	0	16773	257	0

For Manuscript Review

The all-atom clashscore is defined as the number of clashes found per 1000 atoms (including hydrogen atoms). The all-atom clashscore for this structure is 8.

All (257) close contacts within the same asymmetric unit are listed below, sorted by their clash magnitude.

Atom-1	Atom-2	Interatomic distance (Å)	Clash overlap (Å)
1:A:1360:TYR:HB2	2:B:239:GLY:HA3	1.59	0.83
1:A:856:ILE:HD11	1:A:892:VAL:HG11	1.59	0.82
1:A:52:ILE:HG22	1:A:56:LYS:HZ3	1.44	0.82
2:B:109:PRO:HG2	2:B:148:LYS:HB3	1.61	0.81
1:A:1151:PRO:HG3	1:A:1205:VAL:HG21	1.63	0.80
1:A:1124:ASN:ND2	1:A:1174:SER:O	2.17	0.78
1:A:76:GLN:O	1:A:117:ARG:NH1	2.19	0.76
1:A:1245:PRO:O	1:A:1250:ARG:NH2	2.20	0.74
2:B:51:ALA:H	5:B:401:SAH:HA	1.51	0.74
2:B:17:LYS:HE3	3:C:35:A:H5”	1.70	0.73
2:B:51:ALA:HB3	2:B:52:PRO:HD3	1.71	0.72
2:B:159:ARG:NH2	2:B:179:CYS:SG	2.62	0.72
1:A:52:ILE:HG22	1:A:56:LYS:NZ	2.05	0.72
2:B:173:PHE:HE1	2:B:236:VAL:HG21	1.52	0.71
2:B:14:ARG:NH2	3:C:38:A:N7	2.40	0.70
1:A:400:TRP:CD1	1:A:401:GLU:HG3	2.27	0.70
1:A:1219:ARG:NH2	3:C:41:U:O2’	2.25	0.69
2:B:126:ASP:OD1	2:B:161:ARG:NH2	2.25	0.68
1:A:767:HIS:ND1	1:A:767:HIS:O	2.26	0.68
1:A:617:TRP:CD1	1:A:620:LEU:HG	2.28	0.68
2:B:244:TYR:O	2:B:250:TYR:OH	2.09	0.67
2:B:28:LYS:NZ	2:B:191:GLU:OE2	2.26	0.67
3:C:42:A:O2’	3:C:43:A:O5’	2.12	0.67
1:A:1400:GLN:HG3	2:B:244:TYR:HB2	1.76	0.66
1:A:1343:SER:O	1:A:1346:PRO:HD2	1.95	0.66
1:A:1120:THR:HG22	1:A:1174:SER:HB3	1.78	0.66
2:B:75:ASP:OD1	2:B:76:LEU:N	2.29	0.66
1:A:1618:GLU:O	1:A:1622:GLN:NE2	2.30	0.65
1:A:1795:LEU:HD11	1:A:1854:LEU:HD21	1.78	0.65
2:B:116:ASP:HB3	5:B:401:SAH:HN1	1.62	0.65
3:C:47:U:H4’	3:C:48:C:H5’	1.79	0.64
1:A:187:GLN:O	1:A:191:MET:HG2	1.98	0.63
1:A:1692:ASP:O	1:A:1693:HIS:ND1	2.32	0.63
1:A:397:TYR:HD1	1:A:400:TRP:HZ3	1.46	0.62
1:A:1504:PRO:HG2	1:A:1505:TRP:HE3	1.63	0.62
1:A:1505:TRP:HD1	1:A:1507:PHE:HB2	1.65	0.62
1:A:1494:ILE:O	1:A:1497:SER:OG	2.17	0.61

Continued on next page...

For Manuscript Review

Continued from previous page...

Atom-1	Atom-2	Interatomic distance (Å)	Clash overlap (Å)
1:A:1668:CYS:O	1:A:1672:ARG:NH1	2.32	0.61
1:A:80:ASP:HA	1:A:117:ARG:HD3	1.82	0.61
1:A:1155:LEU:HD22	1:A:1164:ILE:HD11	1.82	0.61
1:A:1156:CYS:H	1:A:1162:ALA:HB2	1.66	0.60
1:A:617:TRP:HD1	1:A:620:LEU:H	1.50	0.60
1:A:499:GLU:HA	1:A:502:PHE:CE1	2.37	0.59
1:A:214:ASN:O	1:A:216:TRP:N	2.34	0.59
2:B:103:GLN:HG2	2:B:106:LYS:NZ	2.17	0.59
1:A:1742:GLN:NE2	1:A:1746:ASP:OD1	2.35	0.59
1:A:1055:ARG:NH2	3:C:36:A:OP1	2.35	0.59
1:A:38:LEU:HD11	1:A:54:TYR:HB3	1.85	0.58
1:A:1872:GLN:NE2	1:A:1922:GLU:OE2	2.37	0.58
2:B:181:LYS:NZ	2:B:185:SER:OG	2.37	0.57
2:B:75:ASP:OD2	5:B:401:SAH:O3'	2.22	0.57
1:A:29:ASP:OD1	1:A:37:SER:OG	2.22	0.57
1:A:797:SER:O	1:A:803:LYS:NZ	2.32	0.57
1:A:968:GLN:O	1:A:1104:ARG:NH2	2.37	0.57
1:A:397:TYR:HD1	1:A:400:TRP:CZ3	2.23	0.56
1:A:214:ASN:HA	1:A:217:LYS:HE3	1.87	0.56
1:A:964:SER:O	1:A:967:ILE:HG22	2.06	0.56
2:B:112:LEU:HD12	2:B:152:CYS:HB3	1.87	0.56
1:A:770:GLU:N	1:A:770:GLU:OE1	2.39	0.56
1:A:1752:VAL:O	1:A:1756:MET:HG3	2.06	0.56
1:A:397:TYR:CD1	1:A:400:TRP:HZ3	2.24	0.55
1:A:611:GLN:NE2	1:A:616:THR:O	2.32	0.55
1:A:1505:TRP:CD1	1:A:1507:PHE:HB2	2.41	0.55
1:A:134:TYR:OH	1:A:168:LYS:NZ	2.39	0.55
1:A:101:SER:HA	1:A:104:ASN:HD22	1.71	0.54
2:B:42:VAL:HG13	2:B:111:ASP:HB2	1.89	0.54
1:A:1690:CYS:SG	1:A:1702:VAL:HG11	2.48	0.54
2:B:54:SER:OG	5:B:401:SAH:O	2.26	0.54
1:A:813:LEU:HB2	1:A:816:THR:HG21	1.88	0.54
2:B:14:ARG:NH2	3:C:36:A:H1'	2.23	0.54
1:A:1504:PRO:HG2	1:A:1505:TRP:CE3	2.44	0.53
1:A:1086:VAL:HG13	1:A:1131:LEU:HD21	1.90	0.53
1:A:240:ASP:OD1	1:A:241:ASP:N	2.41	0.52
1:A:1379:ILE:HG21	1:A:1417:TYR:HB2	1.89	0.52
1:A:1308:ASP:OD1	1:A:1309:SER:N	2.42	0.52
1:A:1745:ARG:O	1:A:1749:THR:HG23	2.09	0.52
1:A:1646:ARG:HH12	1:A:1693:HIS:HD2	1.57	0.52
1:A:609:HIS:O	1:A:611:GLN:N	2.42	0.52

Continued on next page...

For Manuscript Review

Continued from previous page...

Atom-1	Atom-2	Interatomic distance (Å)	Clash overlap (Å)
1:A:341:PHE:O	1:A:344:SER:OG	2.27	0.52
1:A:1563:ALA:O	1:A:1566:GLU:HG3	2.09	0.52
1:A:1120:THR:CG2	1:A:1174:SER:HB3	2.40	0.52
1:A:785:VAL:O	1:A:789:GLN:HG2	2.10	0.51
1:A:862:PRO:HG2	1:A:865:LEU:HD13	1.92	0.51
2:B:46:VAL:HB	2:B:113:VAL:HG22	1.92	0.51
1:A:54:TYR:O	1:A:58:ILE:HG13	2.11	0.51
1:A:119:THR:HG23	1:A:158:LEU:HD11	1.92	0.51
1:A:1268:LYS:HA	1:A:1278:ASN:HD21	1.75	0.51
2:B:129:GLU:OE1	2:B:160:GLY:HA3	2.11	0.51
1:A:52:ILE:HG23	1:A:98:VAL:HG11	1.92	0.50
1:A:241:ASP:N	1:A:241:ASP:OD1	2.44	0.50
1:A:1147:LYS:HE2	1:A:1190:GLU:OE2	2.11	0.50
2:B:26:ALA:HB2	2:B:54:SER:HB2	1.94	0.50
1:A:79:LEU:HB2	1:A:117:ARG:NH2	2.26	0.50
2:B:238:CYS:O	2:B:240:ASP:N	2.42	0.50
1:A:1687:ILE:HD11	1:A:1728:ALA:HB1	1.93	0.50
2:B:103:GLN:HG2	2:B:106:LYS:HZ1	1.77	0.50
1:A:389:VAL:HG13	1:A:418:LEU:HD22	1.93	0.49
1:A:53:HIS:HA	1:A:56:LYS:HE2	1.94	0.49
1:A:1043:ASP:N	1:A:1043:ASP:OD1	2.42	0.49
1:A:1104:ARG:O	1:A:1104:ARG:HG2	2.11	0.49
1:A:1335:MET:O	1:A:1335:MET:HG3	2.12	0.49
1:A:881:PRO:HA	1:A:884:VAL:HG12	1.94	0.49
1:A:1160:ARG:O	1:A:1161:SER:HB2	2.12	0.49
1:A:27:PHE:O	1:A:34:ASN:ND2	2.46	0.48
1:A:112:PRO:O	1:A:116:HIS:ND1	2.36	0.48
1:A:148:PHE:CZ	1:A:150:LEU:HB3	2.49	0.48
1:A:890:LEU:HD21	1:A:944:GLU:HB3	1.95	0.48
1:A:1150:ASP:O	1:A:1152:SER:N	2.46	0.48
1:A:392:LEU:HD13	1:A:418:LEU:HD21	1.95	0.48
1:A:1655:ARG:NE	1:A:1704:GLU:OE1	2.45	0.48
1:A:676:PRO:HB2	3:C:26:G:H5”	1.95	0.48
1:A:936:LEU:HB3	1:A:1070:LEU:HD23	1.96	0.48
1:A:1696:THR:HG22	1:A:1700:LEU:HD23	1.95	0.48
2:B:162:ASP:OD1	2:B:162:ASP:N	2.43	0.48
1:A:479:SER:O	1:A:483:GLU:HG2	2.13	0.48
1:A:145:MET:HE3	1:A:145:MET:HA	1.95	0.48
1:A:572:ILE:O	1:A:575:LYS:HB3	2.14	0.48
1:A:720:HIS:O	1:A:722:SER:N	2.46	0.48
1:A:789:GLN:HE22	1:A:825:LYS:HE3	1.78	0.48

Continued on next page...

For Manuscript Review

Continued from previous page...

Atom-1	Atom-2	Interatomic distance (Å)	Clash overlap (Å)
1:A:795:PHE:O	1:A:803:LYS:HE3	2.13	0.48
1:A:1108:ALA:HA	1:A:1111:LEU:HB2	1.95	0.48
1:A:1639:MET:HE1	1:A:1661:ILE:HD11	1.95	0.48
3:C:34:G:O2'	3:C:35:A:H2'	2.14	0.48
1:A:1663:HIS:O	1:A:1667:THR:HG23	2.14	0.48
2:B:13:TYR:CE2	3:C:37:G:H5'	2.49	0.48
1:A:1807:LEU:HD21	1:A:1914:CYS:SG	2.53	0.47
1:A:367:ALA:O	1:A:371:LEU:HB2	2.14	0.47
1:A:869:LEU:HD22	1:A:891:MET:HG2	1.95	0.47
1:A:1425:ASN:OD1	1:A:1426:SER:N	2.47	0.47
1:A:410:GLN:O	1:A:414:MET:HG3	2.15	0.47
1:A:21:LEU:O	1:A:25:LYS:N	2.47	0.47
1:A:1273:GLU:HG2	1:A:1275:SER:H	1.79	0.47
1:A:380:ASP:O	1:A:383:ASN:ND2	2.48	0.47
1:A:1146:ILE:HD11	1:A:1164:ILE:HD13	1.95	0.47
2:B:14:ARG:CZ	3:C:36:A:H1'	2.44	0.47
1:A:145:MET:SD	1:A:155:VAL:HG21	2.54	0.46
1:A:717:THR:OG1	1:A:718:LYS:N	2.45	0.46
1:A:1103:SER:OG	1:A:1107:GLY:O	2.27	0.46
1:A:1425:ASN:HB3	1:A:1428:PHE:HD1	1.80	0.46
1:A:422:HIS:O	1:A:426:VAL:HG22	2.15	0.46
1:A:506:LYS:HD2	1:A:556:TYR:CE2	2.49	0.46
1:A:79:LEU:HD23	1:A:82:LEU:HD12	1.98	0.46
1:A:1177:LYS:O	1:A:1178:LYS:HG2	2.15	0.46
1:A:35:LEU:HD13	1:A:61:LEU:HD12	1.97	0.46
1:A:1756:MET:HG2	1:A:1784:VAL:HG21	1.98	0.46
1:A:18:HIS:HA	1:A:21:LEU:HD23	1.98	0.46
1:A:1463:LEU:O	1:A:1467:THR:HG23	2.15	0.46
2:B:124:LEU:H	2:B:124:LEU:HD23	1.81	0.46
1:A:617:TRP:HD1	1:A:620:LEU:N	2.11	0.46
1:A:636:GLN:NE2	1:A:640:ASP:OD2	2.45	0.45
1:A:89:LEU:HD22	1:A:93:ASN:HD22	1.81	0.45
1:A:1100:LEU:O	1:A:1101:LEU:HD12	2.17	0.45
1:A:1872:GLN:HG3	1:A:1918:LEU:HD13	1.98	0.45
2:B:119:PRO:HG3	2:B:131:MET:HB2	1.98	0.45
2:B:39:PHE:CE2	2:B:59:LEU:HD23	2.52	0.45
1:A:341:PHE:HZ	1:A:392:LEU:HG	1.80	0.45
1:A:1906:ILE:O	1:A:1910:ARG:HG3	2.17	0.45
1:A:80:ASP:CA	1:A:117:ARG:HD3	2.45	0.45
1:A:737:ASN:OD1	1:A:787:ARG:NH2	2.40	0.45
1:A:815:LYS:HE3	1:A:819:HIS:HB2	1.99	0.45

Continued on next page...

For Manuscript Review

Continued from previous page...

Atom-1	Atom-2	Interatomic distance (Å)	Clash overlap (Å)
2:B:172:VAL:HG11	2:B:232:ILE:HG23	1.98	0.45
2:B:203:GLU:HG2	2:B:204:GLY:N	2.32	0.44
1:A:206:GLN:NE2	3:C:56:C:OP1	2.46	0.44
1:A:348:LYS:HE2	1:A:1229:PRO:HB3	2.00	0.44
1:A:361:ALA:O	1:A:364:THR:OG1	2.28	0.44
2:B:99:LYS:HA	2:B:99:LYS:HD3	1.71	0.44
1:A:769:PRO:HA	1:A:772:ARG:HG3	1.99	0.44
2:B:105:PHE:CE2	2:B:110:ALA:HB2	2.52	0.44
2:B:150:GLY:N	2:B:199:TYR:O	2.35	0.44
1:A:352:LEU:HD23	1:A:352:LEU:HA	1.83	0.44
1:A:137:VAL:O	1:A:141:ILE:HG13	2.18	0.44
1:A:1398:PHE:HE2	2:B:249:SER:HA	1.83	0.44
1:A:1199:ASP:OD1	1:A:1199:ASP:N	2.50	0.44
1:A:1706:LEU:HD23	1:A:1706:LEU:HA	1.87	0.44
1:A:236:LYS:HB3	1:A:236:LYS:HE2	1.81	0.43
1:A:616:THR:HG21	1:A:644:LEU:HD21	2.00	0.43
1:A:700:LEU:HD22	1:A:721:PRO:HB3	1.99	0.43
1:A:128:THR:HG21	1:A:137:VAL:HG21	2.00	0.43
1:A:779:LEU:HD23	1:A:813:LEU:HD21	2.00	0.43
1:A:793:GLU:HG2	1:A:1894:GLU:HB3	2.01	0.43
2:B:71:VAL:HB	2:B:85:VAL:HG22	2.00	0.43
1:A:625[B]:ARG:HA	1:A:625[B]:ARG:HD3	1.66	0.43
1:A:706:SER:O	1:A:706:SER:OG	2.34	0.43
2:B:116:ASP:OD1	2:B:156:LYS:HE3	2.18	0.43
1:A:264:HIS:HB3	1:A:265:PRO:HD3	2.01	0.43
1:A:964:SER:N	1:A:965:PRO:HD2	2.34	0.43
1:A:1650:GLN:HE22	1:A:1689:SER:HB3	1.83	0.43
3:C:16:U:H2'	3:C:17:U:O4'	2.19	0.43
1:A:1268:LYS:HA	1:A:1278:ASN:ND2	2.33	0.43
2:B:28:LYS:HG2	2:B:184:SER:HB2	1.99	0.43
1:A:897:MET:HG2	1:A:955:MET:HE1	2.00	0.43
1:A:91:LEU:HD21	1:A:143:SER:OG	2.19	0.43
2:B:91:ASP:HB3	2:B:94:GLN:HG2	2.00	0.43
1:A:55:ILE:HA	1:A:58:ILE:HD12	2.01	0.42
1:A:195:LEU:O	1:A:199:ARG:HG3	2.19	0.42
1:A:1932:LEU:HD23	1:A:1932:LEU:H	1.84	0.42
1:A:652:THR:HG22	1:A:695:GLU:HB3	2.00	0.42
1:A:1381:ASN:O	1:A:1385:THR:HG22	2.19	0.42
1:A:214:ASN:C	1:A:216:TRP:H	2.21	0.42
1:A:1485:GLY:O	1:A:1488:GLU:HG3	2.20	0.42
1:A:1936:ASP:O	1:A:1940:GLU:HG2	2.19	0.42

Continued on next page...

For Manuscript Review

Continued from previous page...

Atom-1	Atom-2	Interatomic distance (Å)	Clash overlap (Å)
1:A:10:GLN:NE2	1:A:11:VAL:O	2.52	0.42
1:A:397:TYR:HA	1:A:400:TRP:CZ3	2.55	0.42
1:A:415:PHE:CE2	1:A:419:LEU:HD11	2.55	0.42
1:A:449:TRP:CZ3	1:A:454:LYS:HE2	2.55	0.42
1:A:459:GLY:O	1:A:463:GLU:HG3	2.20	0.42
1:A:1055:ARG:O	1:A:1059:GLU:HG2	2.20	0.42
1:A:1156:CYS:N	1:A:1162:ALA:HB2	2.33	0.42
1:A:1864:SER:O	1:A:1867:MET:HG2	2.19	0.42
1:A:192:ASN:O	1:A:196:VAL:HG13	2.20	0.42
1:A:1342:LEU:HA	1:A:1342:LEU:HD23	1.86	0.42
1:A:1812:ASP:OD1	1:A:1878:GLN:NE2	2.52	0.42
1:A:1635:LEU:HD11	1:A:1661:ILE:HG13	2.02	0.42
1:A:357:SER:OG	1:A:407:LEU:HD13	2.20	0.42
1:A:364:THR:O	1:A:368:ILE:HG12	2.20	0.42
1:A:869:LEU:HD11	1:A:887:ARG:HH21	1.84	0.42
1:A:529:PRO:O	1:A:533:ILE:HG12	2.20	0.41
1:A:631:ILE:HD12	1:A:631:ILE:HA	1.90	0.41
1:A:1635:LEU:HD11	1:A:1661:ILE:CG1	2.50	0.41
2:B:137:LEU:HD12	2:B:137:LEU:HA	1.90	0.41
1:A:1851:LEU:HD23	1:A:1851:LEU:HA	1.83	0.41
1:A:52:ILE:C	1:A:56:LYS:HZ3	2.24	0.41
1:A:506:LYS:HE2	1:A:518:TRP:CH2	2.55	0.41
1:A:1146:ILE:HD13	1:A:1146:ILE:HA	1.89	0.41
1:A:1433:THR:O	1:A:1436:THR:HG22	2.20	0.41
1:A:1466:LEU:HD23	1:A:1466:LEU:HA	1.87	0.41
1:A:1623:THR:HG22	1:A:1629:LEU:O	2.21	0.41
1:A:1671:ASN:HA	1:A:1674:LEU:HG	2.01	0.41
1:A:1162:ALA:C	1:A:1164:ILE:H	2.24	0.41
2:B:13:TYR:HE2	3:C:37:G:H5'	1.85	0.41
2:B:151:GLY:H	2:B:199:TYR:HB3	1.86	0.41
1:A:321:GLN:H	1:A:321:GLN:HG3	1.73	0.41
1:A:1099:HIS:C	1:A:1101:LEU:H	2.24	0.41
1:A:1384:ARG:HG2	1:A:1438:CYS:SG	2.61	0.41
1:A:27:PHE:HB3	1:A:74:THR:HG22	2.03	0.41
1:A:35:LEU:HD12	1:A:38:LEU:HB3	2.03	0.41
1:A:159:LEU:HD21	1:A:208:VAL:HG11	2.03	0.41
1:A:933:LYS:HE3	1:A:933:LYS:HB3	1.91	0.41
1:A:1582:PRO:HA	1:A:1585:CYS:SG	2.61	0.41
1:A:1117:VAL:O	1:A:1121:GLU:HG3	2.21	0.41
1:A:1131:LEU:HD23	1:A:1131:LEU:HA	1.86	0.41
2:B:91:ASP:O	2:B:97:THR:OG1	2.24	0.41

Continued on next page...

For Manuscript Review

Continued from previous page...

Atom-1	Atom-2	Interatomic distance (Å)	Clash overlap (Å)
1:A:175:GLU:O	1:A:178:ARG:HG2	2.21	0.40
1:A:348:LYS:HB2	1:A:348:LYS:HE3	1.79	0.40
1:A:1369:ALA:O	1:A:1372:PRO:HD2	2.21	0.40
2:B:170:LEU:HD23	2:B:170:LEU:HA	1.88	0.40
1:A:59:VAL:HB	1:A:60:PRO:HD3	2.02	0.40
1:A:869:LEU:H	1:A:869:LEU:HD23	1.86	0.40
1:A:941:LEU:O	1:A:944:GLU:N	2.52	0.40
1:A:1710:THR:HG22	1:A:1714:LEU:HD12	2.03	0.40
1:A:449:TRP:O	1:A:455:TYR:OH	2.25	0.40
1:A:1646:ARG:HH12	1:A:1693:HIS:CD2	2.38	0.40
1:A:1219:ARG:O	3:C:42:A:H5”	2.21	0.40
1:A:767:HIS:O	1:A:767:HIS:CG	2.74	0.40
1:A:834:LEU:HD23	1:A:834:LEU:HA	1.89	0.40
1:A:1307:VAL:HG11	1:A:1356:HIS:HB2	2.03	0.40
2:B:174:PHE:CG	2:B:196:CYS:HB3	2.57	0.40

There are no symmetry-related clashes.

5.3 Torsion angles (i)

5.3.1 Protein backbone (i)

In the following table, the Percentiles column shows the percent Ramachandran outliers of the chain as a percentile score with respect to all PDB entries followed by that with respect to all EM entries.

The Analysed column shows the number of residues for which the backbone conformation was analysed, and the total number of residues.

Mol	Chain	Analysed	Favoured	Allowed	Outliers	Percentiles	
1	A	1765/1981 (89%)	1672 (95%)	92 (5%)	1 (0%)	48	67
2	B	220/347 (63%)	208 (94%)	12 (6%)	0	100	100
All	All	1985/2328 (85%)	1880 (95%)	104 (5%)	1 (0%)	50	67

All (1) Ramachandran outliers are listed below:

Mol	Chain	Res	Type
1	A	215	LEU

For Manuscript Review

5.3.2 Protein sidechains (i)

In the following table, the Percentiles column shows the percent sidechain outliers of the chain as a percentile score with respect to all PDB entries followed by that with respect to all EM entries.

The Analysed column shows the number of residues for which the sidechain conformation was analysed, and the total number of residues.

Mol	Chain	Analysed	Rotameric	Outliers	Percentiles	
1	A	1601/1775 (90%)	1599 (100%)	2 (0%)	92	97
2	B	185/296 (62%)	184 (100%)	1 (0%)	86	94
All	All	1786/2071 (86%)	1783 (100%)	3 (0%)	91	97

All (3) residues with a non-rotameric sidechain are listed below:

Mol	Chain	Res	Type
1	A	880	ARG
1	A	1106	ARG
2	B	148	LYS

Sometimes sidechains can be flipped to improve hydrogen bonding and reduce clashes. All (3) such sidechains are listed below:

Mol	Chain	Res	Type
1	A	41	HIS
1	A	104	ASN
1	A	1693	HIS

5.3.3 RNA (i)

Mol	Chain	Analysed	Backbone Outliers	Pucker Outliers
3	C	71/76 (93%)	12 (16%)	0

All (12) RNA backbone outliers are listed below:

Mol	Chain	Res	Type
3	C	16	U
3	C	18	G
3	C	20	G
3	C	21	A
3	C	32	C
3	C	36	A

Continued on next page...

For Manuscript Review

Continued from previous page...

Mol	Chain	Res	Type
3	C	37	G
3	C	41	U
3	C	43	A
3	C	47	U
3	C	53	G
3	C	69	C

There are no RNA pucker outliers to report.

5.4 Non-standard residues in protein, DNA, RNA chains (i)

There are no non-standard protein/DNA/RNA residues in this entry.

5.5 Carbohydrates (i)

There are no oligosaccharides in this entry.

5.6 Ligand geometry (i)

Of 9 ligands modelled in this entry, 8 are monoatomic - leaving 1 for Mogul analysis.

In the following table, the Counts columns list the number of bonds (or angles) for which Mogul statistics could be retrieved, the number of bonds (or angles) that are observed in the model and the number of bonds (or angles) that are defined in the Chemical Component Dictionary. The Link column lists molecule types, if any, to which the group is linked. The Z score for a bond length (or angle) is the number of standard deviations the observed value is removed from the expected value. A bond length (or angle) with $|Z| > 2$ is considered an outlier worth inspection. RMSZ is the root-mean-square of all Z scores of the bond lengths (or angles).

Mol	Type	Chain	Res	Link	Bond lengths			Bond angles		
					Counts	RMSZ	$\#\|Z\| > 2$	Counts	RMSZ	$\#\|Z\| > 2$
5	SAH	B	401	-	24,28,28	1.03	2 (8%)	25,40,40	1.26	2 (8%)

In the following table, the Chirals column lists the number of chiral outliers, the number of chiral centers analysed, the number of these observed in the model and the number defined in the Chemical Component Dictionary. Similar counts are reported in the Torsion and Rings columns. '-' means no outliers of that kind were identified.

Mol	Type	Chain	Res	Link	Chirals	Torsions	Rings
5	SAH	B	401	-	-	5/11/31/31	0/3/3/3

For Manuscript Review

All (2) bond length outliers are listed below:

Mol	Chain	Res	Type	Atoms	Z	Observed(Å)	Ideal(Å)
5	B	401	SAH	O3'-C3'	-2.70	1.36	1.43
5	B	401	SAH	O2'-C2'	-2.01	1.38	1.43

All (2) bond angle outliers are listed below:

Mol	Chain	Res	Type	Atoms	Z	Observed(^{o})	Ideal(^{o})
5	B	401	SAH	O4'-C1'-C2'	-3.27	102.15	106.93
5	B	401	SAH	O3'-C3'-C4'	-2.42	104.06	111.05

There are no chirality outliers.

All (5) torsion outliers are listed below:

Mol	Chain	Res	Type	Atoms
5	B	401	SAH	C4'-C5'-SD-CG
5	B	401	SAH	O4'-C4'-C5'-SD
5	B	401	SAH	C3'-C4'-C5'-SD
5	B	401	SAH	OXT-C-CA-CB
5	B	401	SAH	O-C-CA-CB

There are no ring outliers.

1 monomer is involved in 4 short contacts:

Mol	Chain	Res	Type	Clashes	Symm-Clashes
5	B	401	SAH	4	0

The following is a two-dimensional graphical depiction of Mogul quality analysis of bond lengths, bond angles, torsion angles, and ring geometry for all instances of the Ligand of Interest. In addition, ligands with molecular weight > 250 and outliers as shown on the validation Tables will also be included. For torsion angles, if less then 5% of the Mogul distribution of torsion angles is within 10 degrees of the torsion angle in question, then that torsion angle is considered an outlier. Any bond that is central to one or more torsion angles identified as an outlier by Mogul will be highlighted in the graph. For rings, the root-mean-square deviation (RMSD) between the ring in question and similar rings identified by Mogul is calculated over all ring torsion angles. If the average RMSD is greater than 60 degrees and the minimal RMSD between the ring in question and any Mogul-identified rings is also greater than 60 degrees, then that ring is considered an outlier. The outliers are highlighted in purple. The color gray indicates Mogul did not find sufficient equivalents in the CSD to analyse the geometry.

For Manuscript Review

Ligand SAH B 401

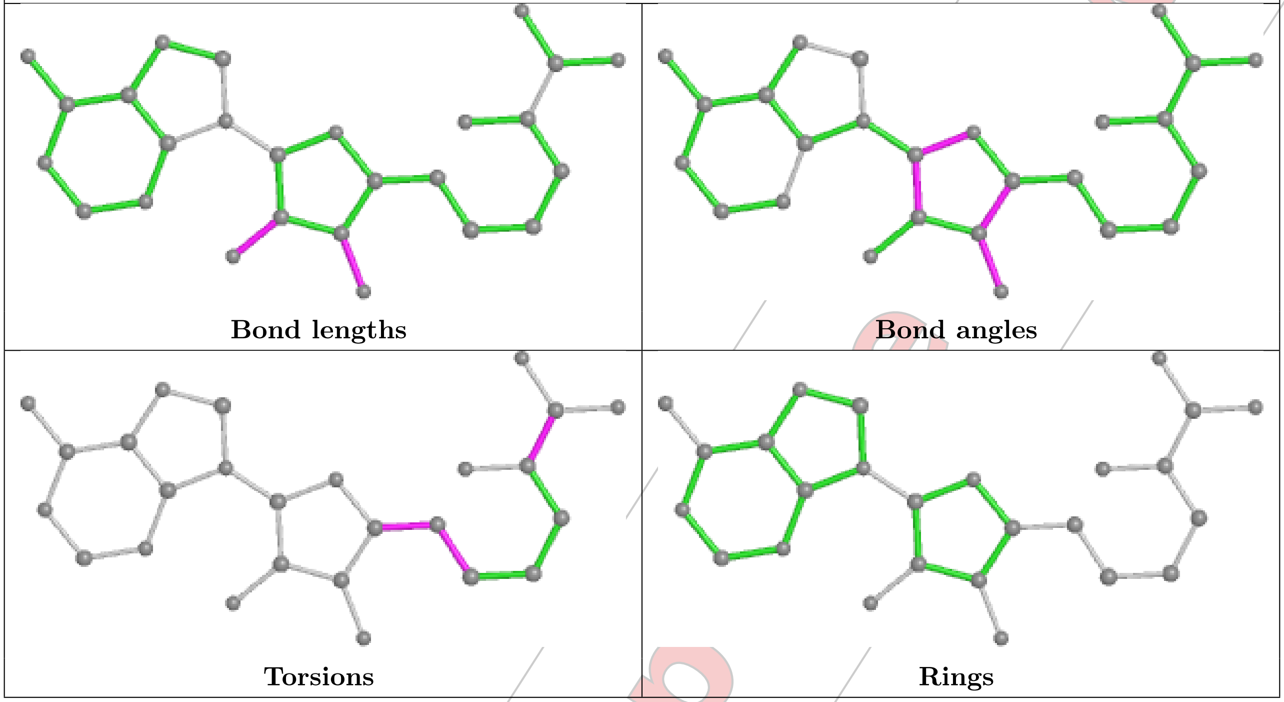

5.7 Other polymers (i)

There are no such residues in this entry.

5.8 Polymer linkage issues (i)

There are no chain breaks in this entry.

For Manuscript Review

6 Map visualisation (i)

This section contains visualisations of the EMDB entry EMD-38859. These allow visual inspection of the internal detail of the map and identification of artifacts.

Images derived from a raw map, generated by summing the deposited half-maps, are presented below the corresponding image components of the primary map to allow further visual inspection and comparison with those of the primary map.

6.1 Orthogonal projections (i)

6.1.1 Primary map

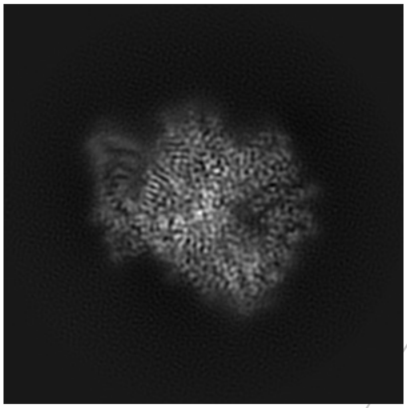

X

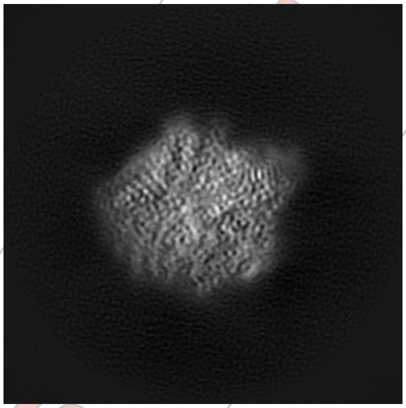

Y

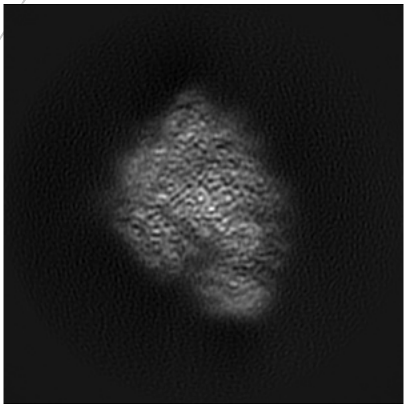

Z

6.1.2 Raw map

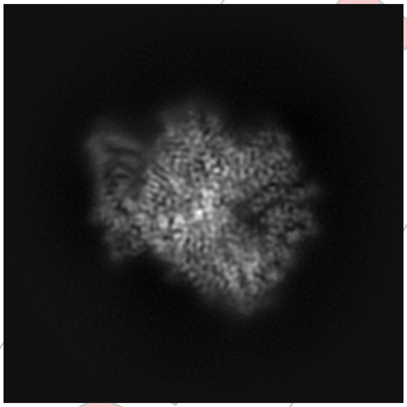

X

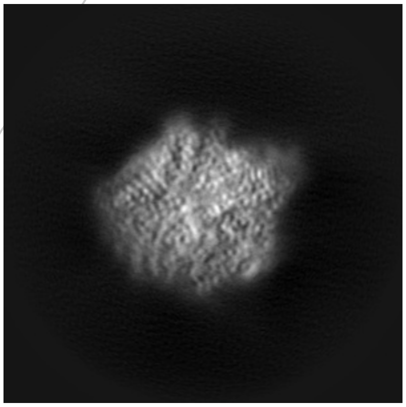

Y

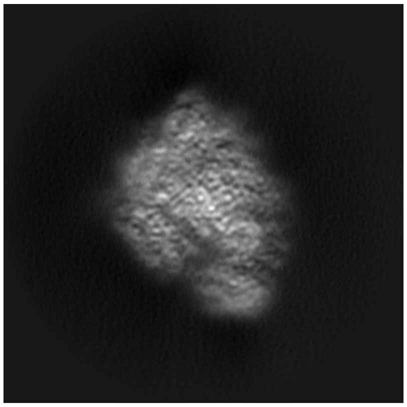

Z

The images above show the map projected in three orthogonal directions.

For Manuscript Review

6.2 Central slices (i)

6.2.1 Primary map

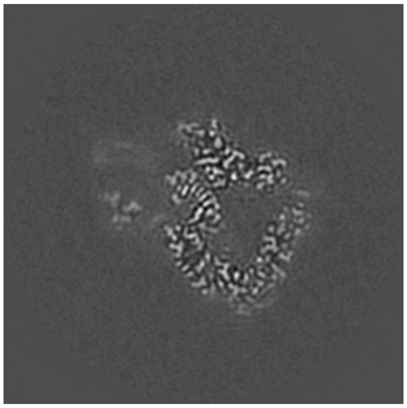
X Index: 130

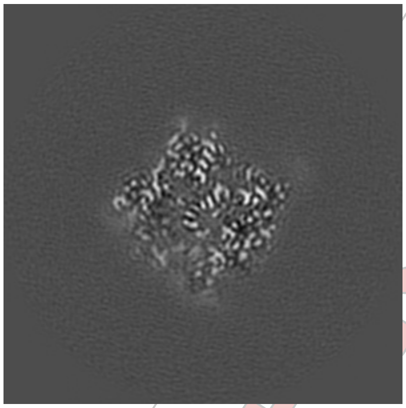
Y Index: 130

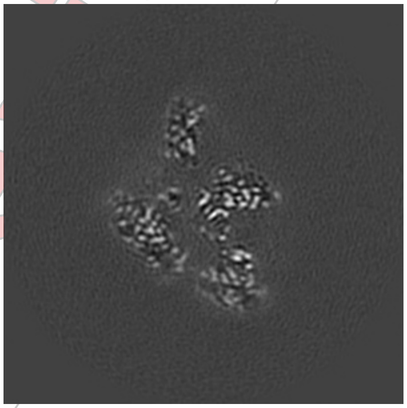
Z Index: 130

6.2.2 Raw map

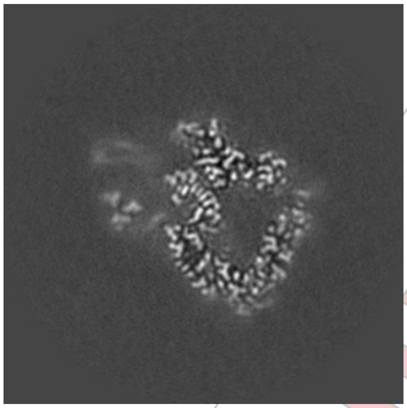
X Index: 130

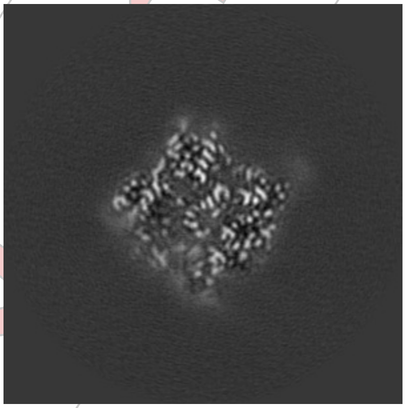
Y Index: 130

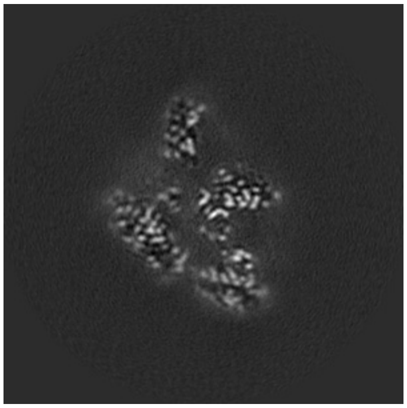
Z Index: 130

The images above show central slices of the map in three orthogonal directions.

For Manuscript Review

6.3 Largest variance slices (i)

6.3.1 Primary map

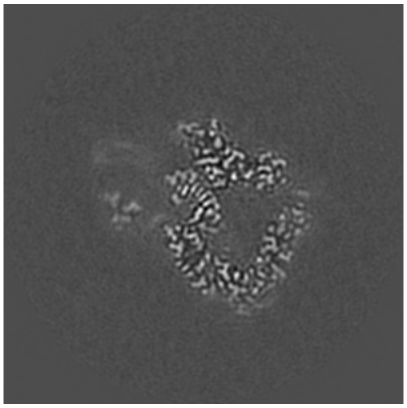
X Index: 130

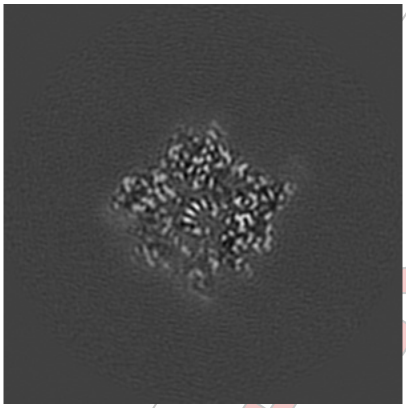
Y Index: 132

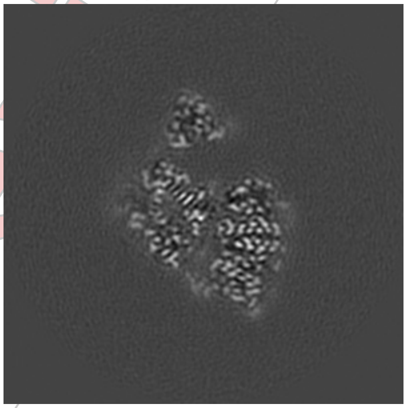
Z Index: 118

6.3.2 Raw map

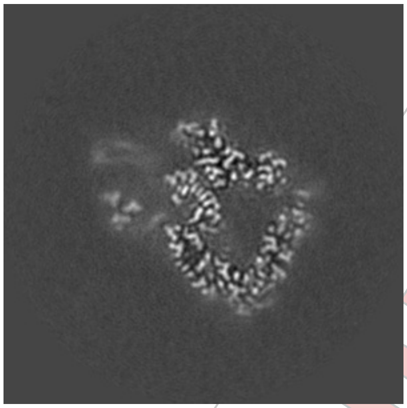
X Index: 130

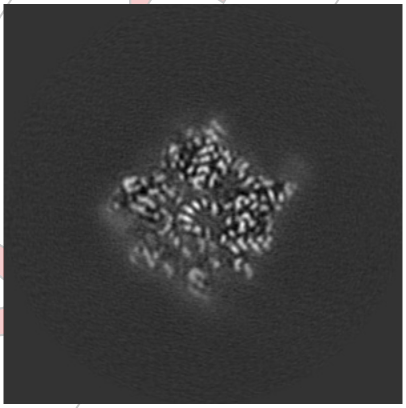
Y Index: 133

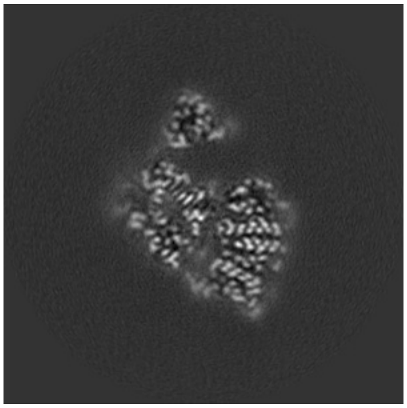
Z Index: 118

The images above show the largest variance slices of the map in three orthogonal directions.

For Manuscript Review

6.4 Orthogonal standard-deviation projections (False-color) (i)

6.4.1 Primary map

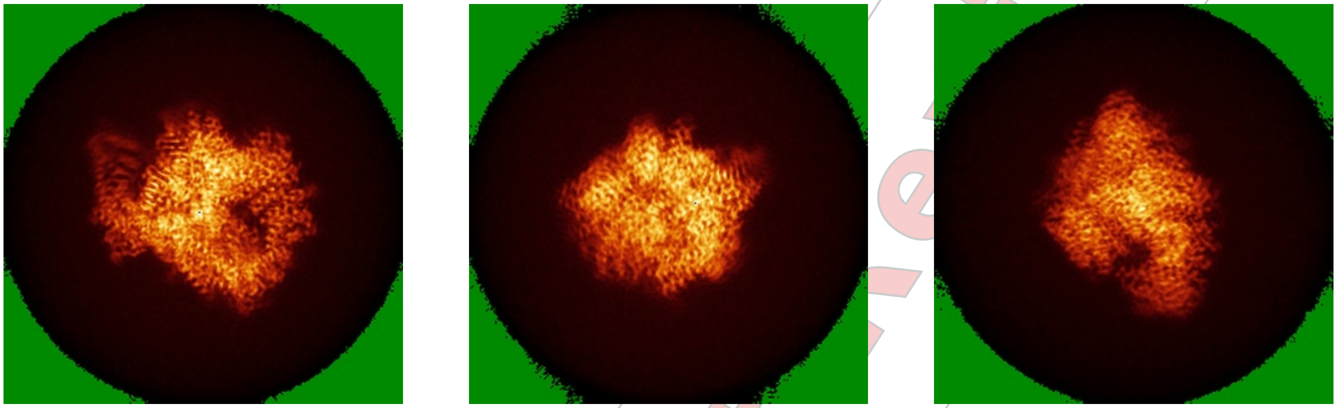

X Y Z

6.4.2 Raw map

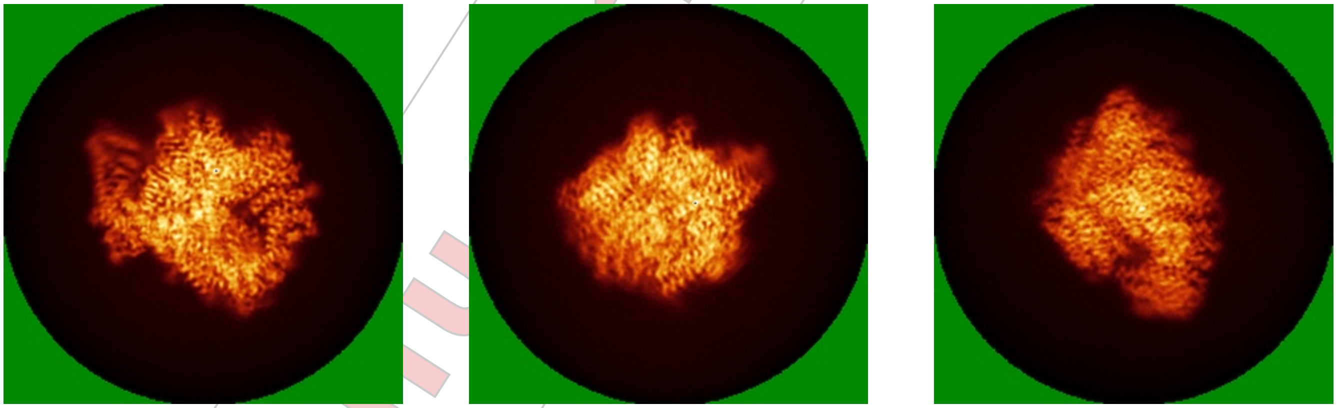

X Y Z

The images above show the map standard deviation projections with false color in three orthogonal directions. Minimum values are shown in green, max in blue, and dark to light orange shades represent small to large values respectively.

For Manuscript Review

6.5 Orthogonal surface views (i)

6.5.1 Primary map

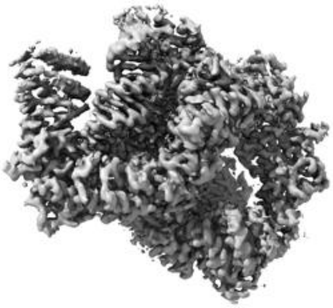

X

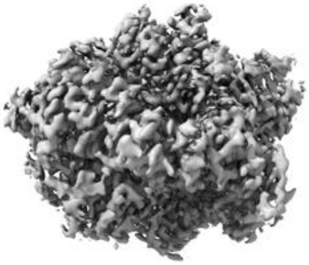

Y

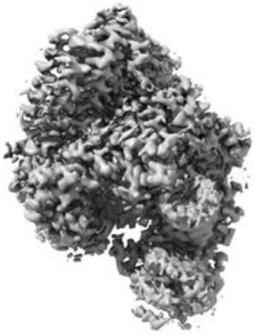

Z

The images above show the 3D surface view of the map at the recommended contour level 0.0126. These images, in conjunction with the slice images, may facilitate assessment of whether an appropriate contour level has been provided.

6.5.2 Raw map

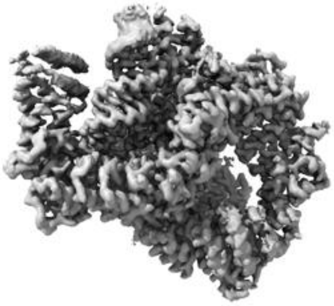

X

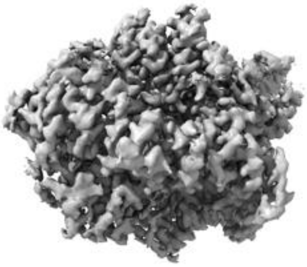

Y

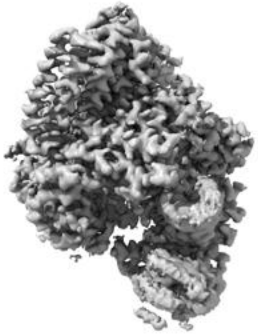

Z

These images show the 3D surface of the raw map. The raw map's contour level was selected so that its surface encloses the same volume as the primary map does at its recommended contour level.

6.6 Mask visualisation (i)

This section was not generated. No masks/segmentation were deposited.

For Manuscript Review

7 Map analysis (i)

This section contains the results of statistical analysis of the map.

7.1 Map-value distribution (i)

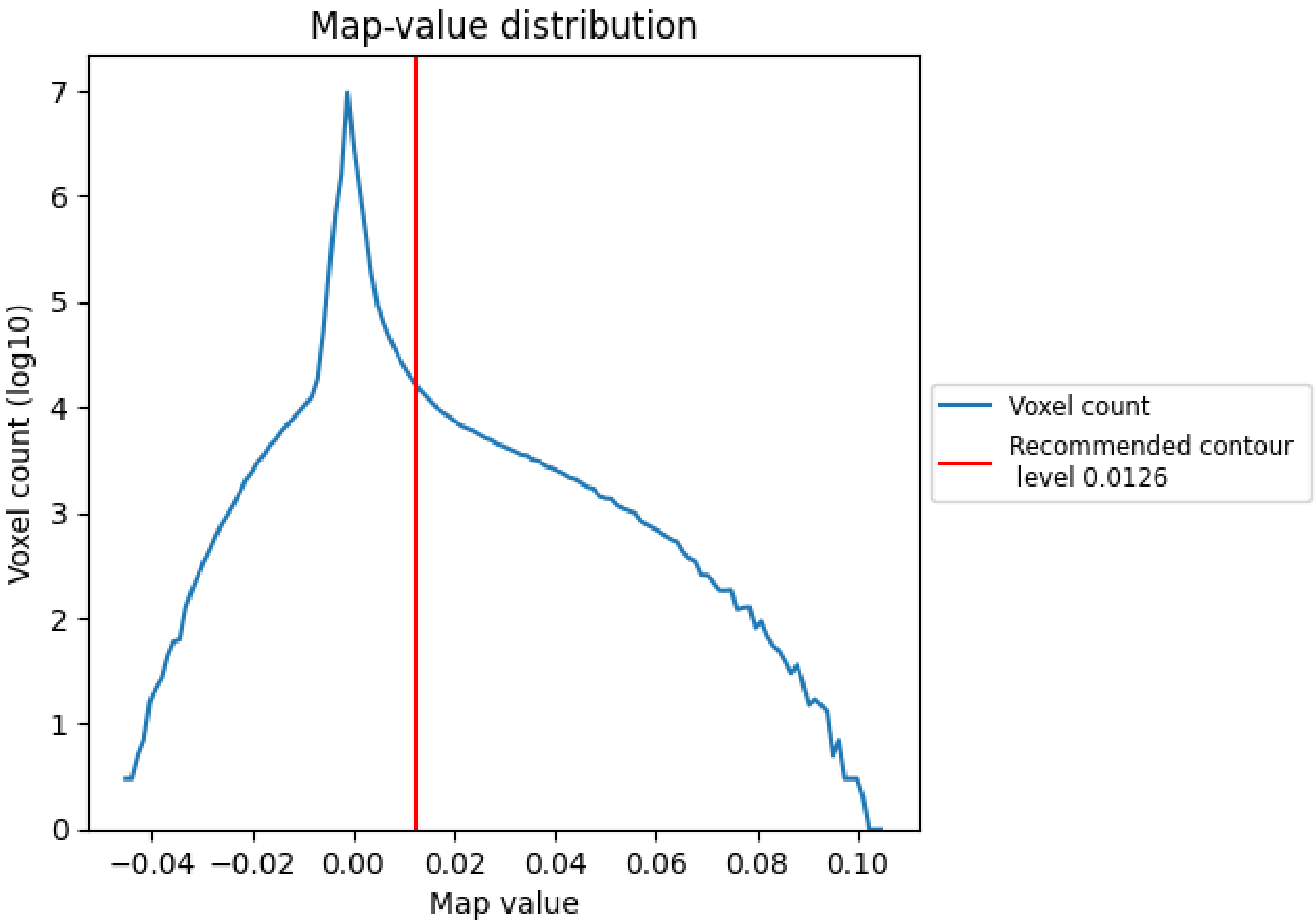

The map-value distribution is plotted in 128 intervals along the x-axis. The y-axis is logarithmic. A spike in this graph at zero usually indicates that the volume has been masked.

For Manuscript Review

7.2 Volume estimate (i)

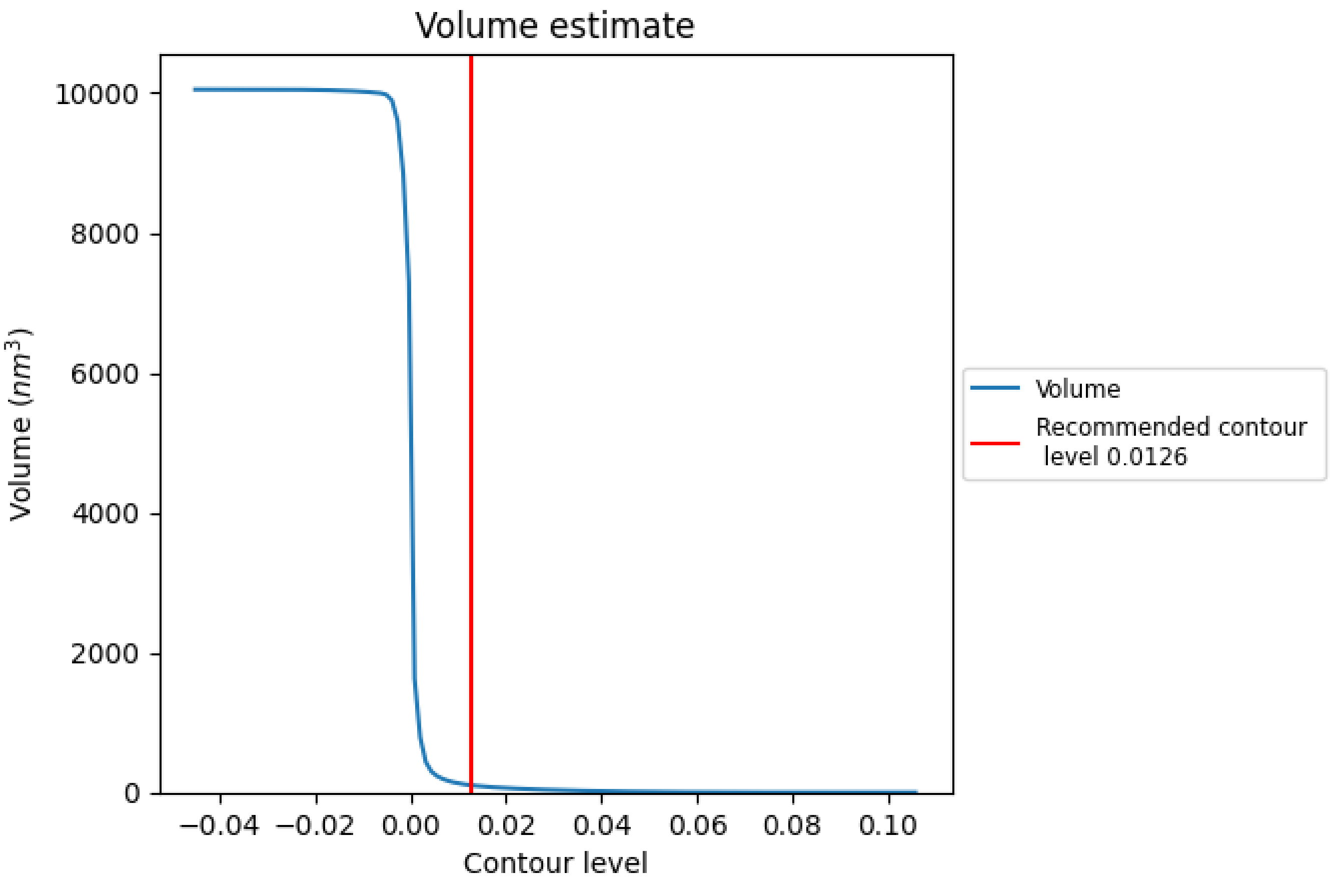

The volume at the recommended contour level is 103 nm^3; this corresponds to an approximate mass of 93 kDa.

The volume estimate graph shows how the enclosed volume varies with the contour level. The recommended contour level is shown as a vertical line and the intersection between the line and the curve gives the volume of the enclosed surface at the given level.

For Manuscript Review

7.3 Rotationally averaged power spectrum (i)

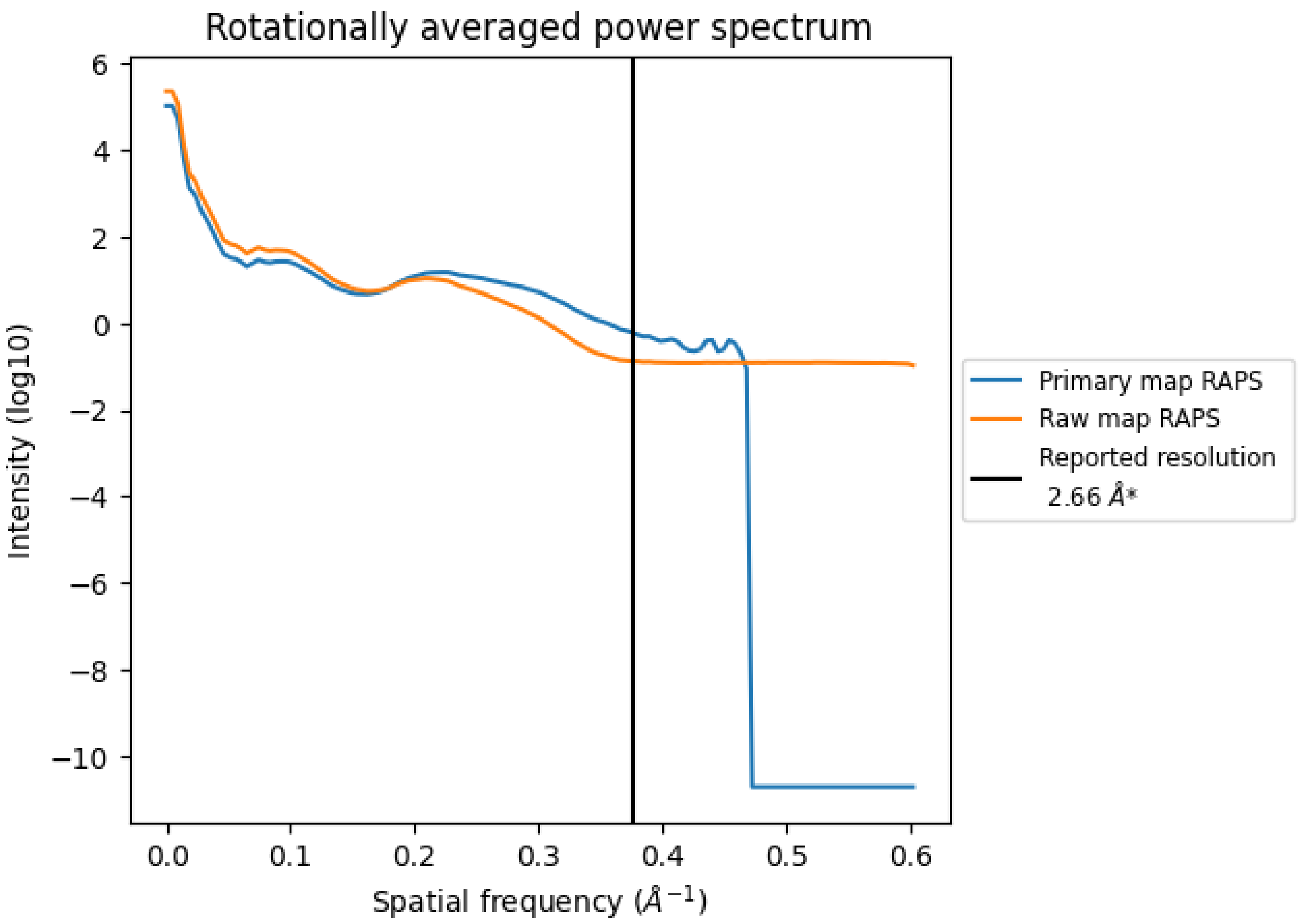

*Reported resolution corresponds to spatial frequency of 0.376 Å^{-1}

For Manuscript Review

8 Fourier-Shell correlation (i)

Fourier-Shell Correlation (FSC) is the most commonly used method to estimate the resolution of single-particle and subtomogram-averaged maps. The shape of the curve depends on the imposed symmetry, mask and whether or not the two 3D reconstructions used were processed from a common reference. The reported resolution is shown as a black line. A curve is displayed for the half-bit criterion in addition to lines showing the 0.143 gold standard cut-off and 0.5 cut-off.

8.1 FSC (i)

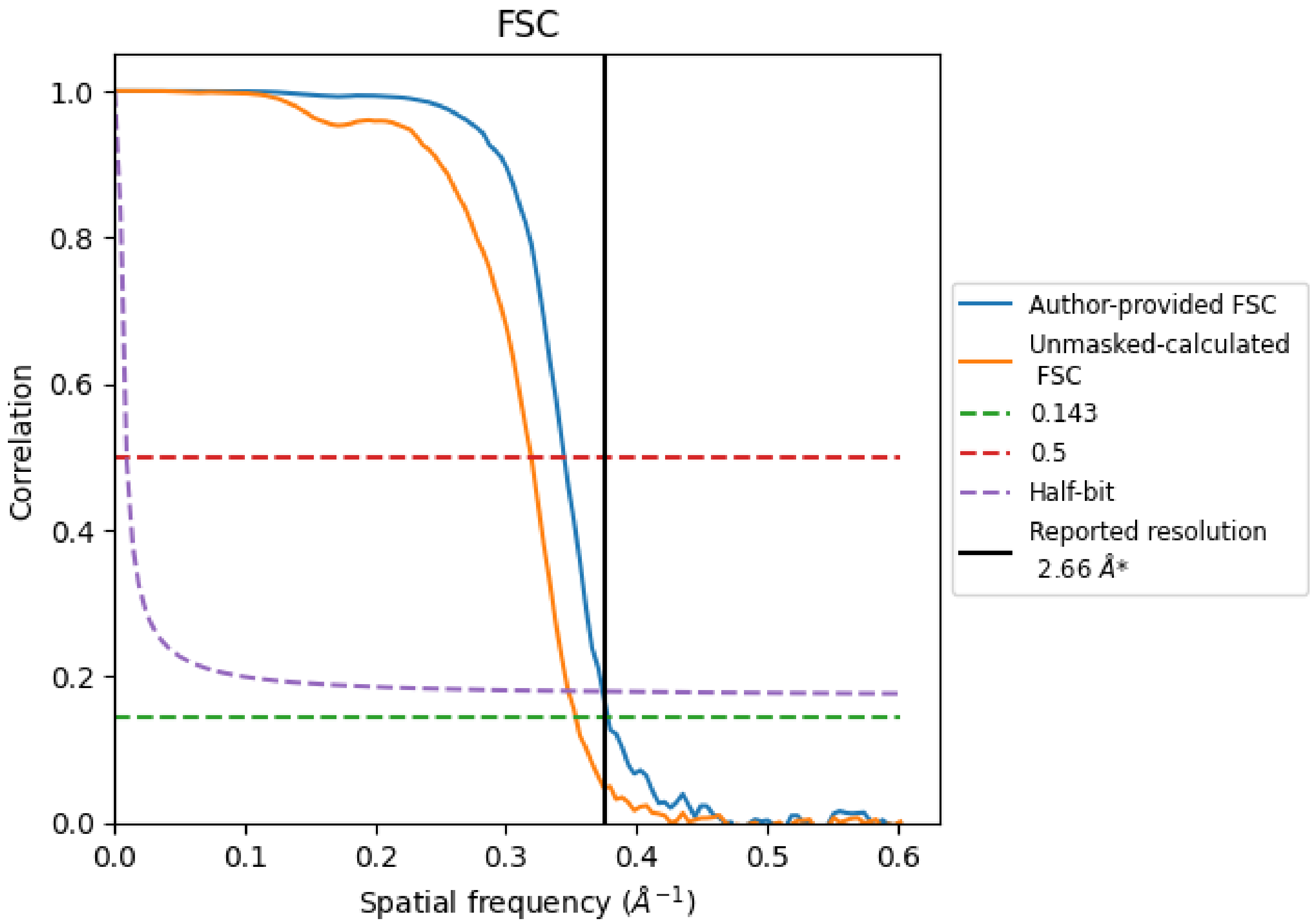

*Reported resolution corresponds to spatial frequency of 0.376 Å^{-1}

For Manuscript Review

8.2 Resolution estimates (i)

Resolution estimate (Å)	Estimation criterion (FSC cut-off)		
	0.143	0.5	Half-bit
Reported by author	2.66	-	-
Author-provided FSC curve	2.64	2.90	2.67
Unmasked-calculated*	2.83	3.13	2.87

*Resolution estimate based on FSC curve calculated by comparison of deposited half-maps.

For Manuscript Review

9 Map-model fit (i)

This section contains information regarding the fit between EMDB map EMD-38859 and PDB model 8Y2O. Per-residue inclusion information can be found in section 3 on page 6.

9.1 Map-model overlay (i)

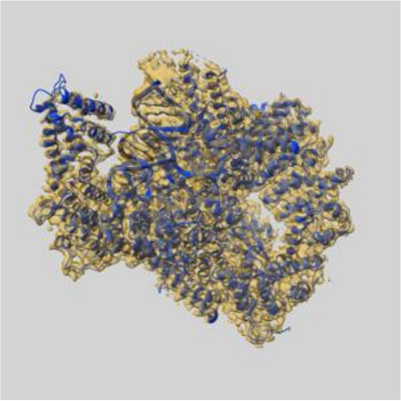

X

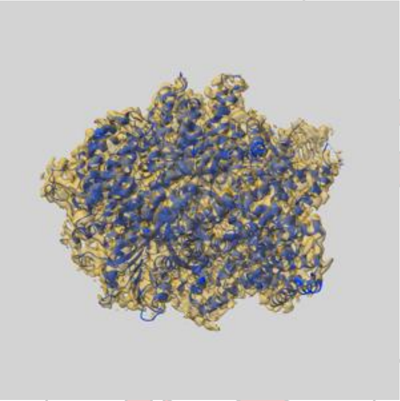

Y

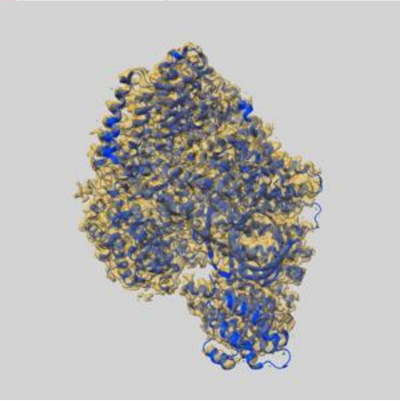

Z

The images above show the 3D surface view of the map at the recommended contour level 0.0126 at 50% transparency in yellow overlaid with a ribbon representation of the model coloured in blue. These images allow for the visual assessment of the quality of fit between the atomic model and the map.

For Manuscript Review

9.2 Q-score mapped to coordinate model (i)

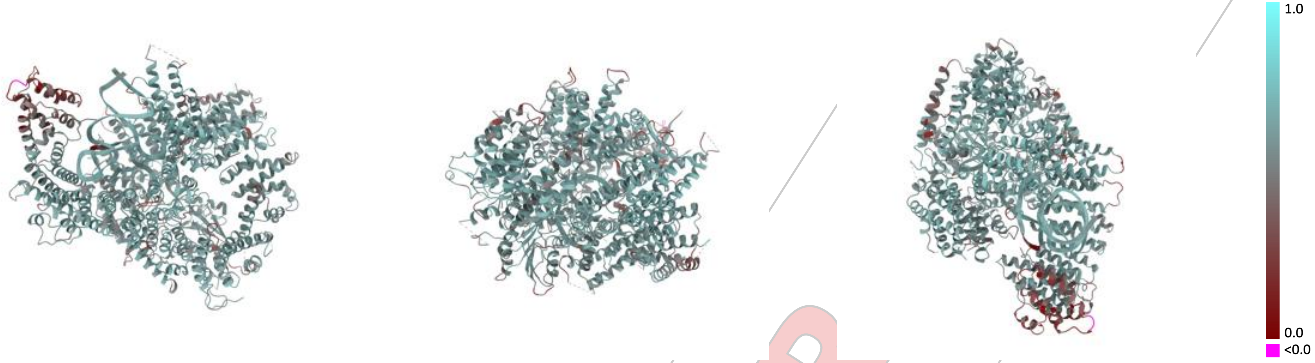

X Y Z

The images above show the model with each residue coloured according its Q-score. This shows their resolvability in the map with higher Q-score values reflecting better resolvability. Please note: Q-score is calculating the resolvability of atoms, and thus high values are only expected at resolutions at which atoms can be resolved. Low Q-score values may therefore be expected for many entries.

9.3 Atom inclusion mapped to coordinate model (i)

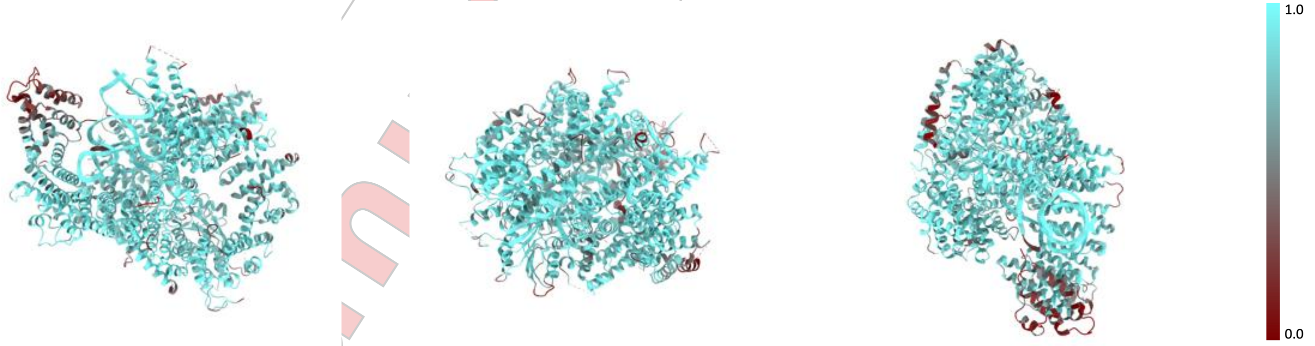

X Y Z

The images above show the model with each residue coloured according to its atom inclusion. This shows to what extent they are inside the map at the recommended contour level (0.0126).

For Manuscript Review

9.4 Atom inclusion (i)

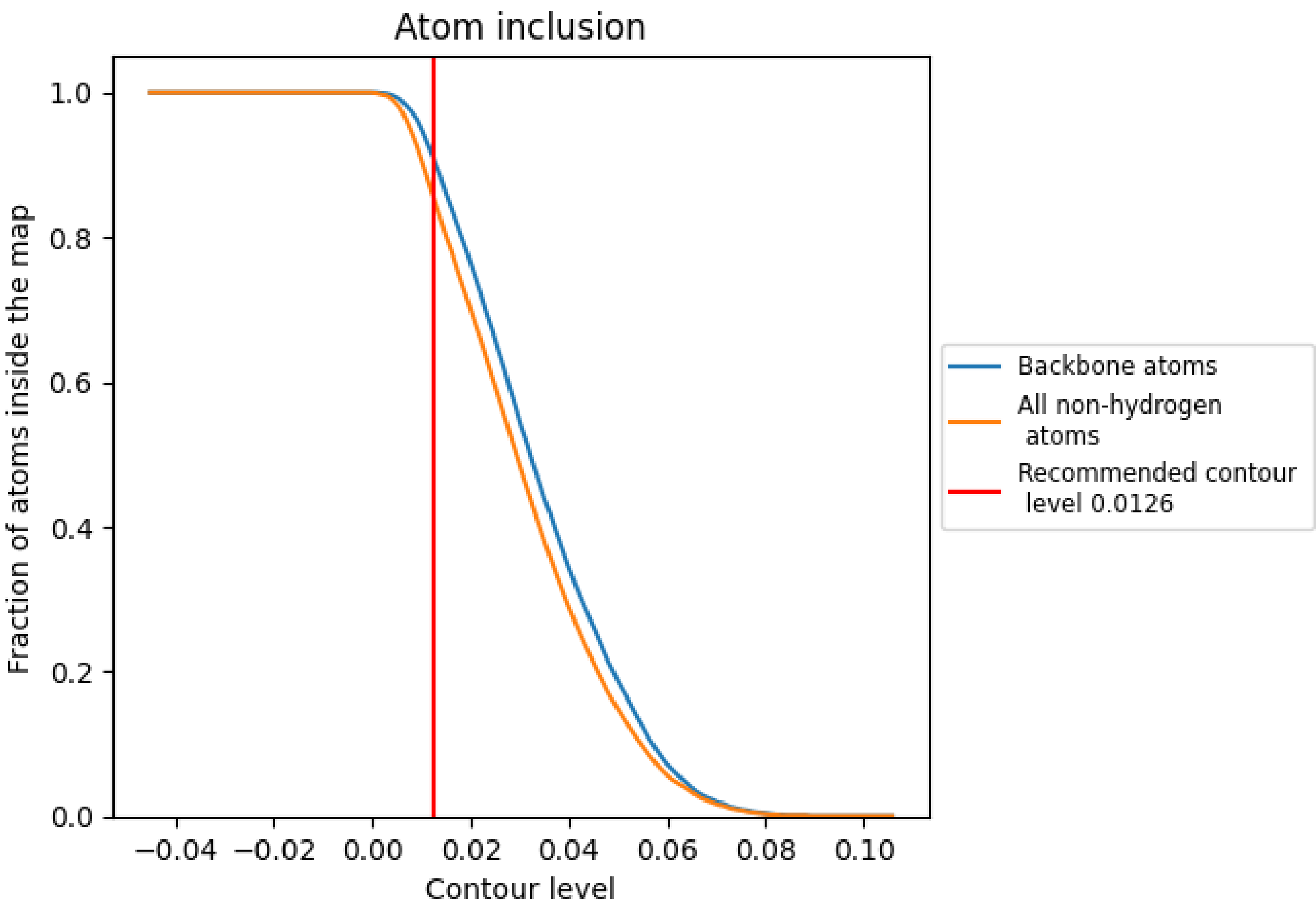

At the recommended contour level, 91% of all backbone atoms, 85% of all non-hydrogen atoms, are inside the map.

For Manuscript Review

9.5 Map-model fit summary (i)

The table lists the average atom inclusion at the recommended contour level (0.0126) and Q-score for the entire model and for each chain.

Chain	Atom inclusion	Q-score
All	0.8540	0.5820
A	0.8420	0.5770
B	0.8500	0.5820
C	0.9590	0.6230

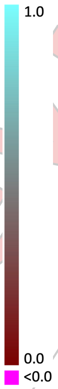

For Manuscript Review
