# Supplementary material for: Structural insights into tRNA recognition of the human FTSJ1-THADA complex
Source: Commun Biol. 2025 Jun 7;8:893. doi: 10.1038/s42003-025-08278-3 (PMC12145424; doi:10.1038/s42003-025-08278-3)
Supplement: Supplementary file 16 — Reporting Summary [file 42003_2025_8278_MOESM16_ESM.pdf]

nature portfolio

Corresponding author(s): M. Shirouzu

Last updated by author(s): May 14, 2025

Reporting Summary

Nature Portfolio wishes to improve the reproducibility of the work that we publish. This form provides structure for consistency and transparency in reporting. For further information on Nature Portfolio policies, see our Editorial Policies and the Editorial Policy Checklist.

Statistics

For all statistical analyses, confirm that the following items are present in the figure legend, table legend, main text, or Methods section.

n/a	Confirmed	
☐	☒	The exact sample size (*n*) for each experimental group/condition, given as a discrete number and unit of measurement
☐	☒	A statement on whether measurements were taken from distinct samples or whether the same sample was measured repeatedly
☐	☒	The statistical test(s) used AND whether they are one- or two-sided<br>*Only common tests should be described solely by name; describe more complex techniques in the Methods section.*
☒	☐	A description of all covariates tested
☒	☐	A description of any assumptions or corrections, such as tests of normality and adjustment for multiple comparisons
☐	☒	A full description of the statistical parameters including central tendency (e.g. means) or other basic estimates (e.g. regression coefficient) AND variation (e.g. standard deviation) or associated estimates of uncertainty (e.g. confidence intervals)
☐	☒	For null hypothesis testing, the test statistic (e.g. *F*, *t*, *r*) with confidence intervals, effect sizes, degrees of freedom and *P* value noted<br>*Give P values as exact values whenever suitable.*
☒	☐	For Bayesian analysis, information on the choice of priors and Markov chain Monte Carlo settings
☒	☐	For hierarchical and complex designs, identification of the appropriate level for tests and full reporting of outcomes
☒	☐	Estimates of effect sizes (e.g. Cohen's *d*, Pearson's *r*), indicating how they were calculated

Our web collection on statistics for biologists contains articles on many of the points above.

Software and code

Policy information about availability of computer code

Data collection	Automated cryo-EM data acquisition was performed by EPU 2.9 software (Thermo Fisher Scientific) on a Krios G4 transmission electron microscope (FEI) equipped with a K3 direct electron detector (Gatan).<br>The methyltransferase activity of FTSJ1-THADA mutants was measured using FlexStation3 (Molecular Devices).
Data analysis	Canvas X (version 20) and ChemDraw (20.1) were used to create figures.<br>UCSF Chimera (version 1.15) and UCSF ChimeraX (version 1.2) were used to analyze and prepare figures of cryo-EM maps and atomic models.<br>Phenix (1. 19. 2) and Coot (version 0.9.4) were used for model building.<br>RELION 3.1.3 and crYOLO (1.9.1) were used for cryo-EM image processing.

For manuscripts utilizing custom algorithms or software that are central to the research but not yet described in published literature, software must be made available to editors and reviewers. We strongly encourage code deposition in a community repository (e.g. GitHub). See the Nature Portfolio guidelines for submitting code & software for further information.

Data

Policy information about availability of data

All manuscripts must include a data availability statement. This statement should provide the following information, where applicable:

- Accession codes, unique identifiers, or web links for publicly available datasets
- A description of any restrictions on data availability
- For clinical datasets or third party data, please ensure that the statement adheres to our policy

Publicly available datasets from Protein Data Bank (7Y7F and 6JPL) were used for atomic model building and comparison.
Cryo-EM maps and atomic coordinates of the reported structures were deposited in Electron Microscopy Data Bank (EMDB) and Protein Data Bank, respectively, with the following accession codes; EMD-38859 and 8Y2O (FTSJ1-THADA with tRNA); EMD-61701 (FTSJ1-THADA, Class 1); EMD-61702 (FTSJ1-THADA, Class 2a); EMD-61703 (FTSJ1-THADA, Class 2b); EMD-61704 (FTSJ1-THADA, Class 2c); EMD-61705 (FTSJ1-THADA, Class 2d).

Research involving human participants, their data, or biological material

Policy information about studies with human participants or human data. See also policy information about sex, gender (identity/presentation), and sexual orientation and race, ethnicity and racism.

Reporting on sex and gender	This study does not involve human research.
Reporting on race, ethnicity, or other socially relevant groupings	This study does not involve human research.
Population characteristics	This study does not involve human research.
Recruitment	This study does not involve human research.
Ethics oversight	This study does not involve human research.

Note that full information on the approval of the study protocol must also be provided in the manuscript.

Field-specific reporting

Please select the one below that is the best fit for your research. If you are not sure, read the appropriate sections before making your selection.

☒ Life sciences ☐ Behavioural & social sciences ☐ Ecological, evolutionary & environmental sciences

For a reference copy of the document with all sections, see nature.com/documents/nr-reporting-summary-flat.pdf

Life sciences study design

All studies must disclose on these points even when the disclosure is negative.

Sample size	No sample size calculation was conducted. The sample size for cryo-EM analysis was determined on the basis of a overnight data collection to obtain sufficient number of particles and achieve high resolution of the complex.
Data exclusions	Particles in the poorly resolved classes after classification were excluded as is the standard practice in cryo-EM analysis.
Replication	The methyltransferase assay of FTSJ1-THADA was performed in triplicate (n=3) to confirm the exact values of the methyltransferase activity and all attempts were successful with consistent results as shown in the figures. Although cryo-EM analysis was not replicated, the atomic models were generated from thousands of micrographs.
Randomization	For cryo-EM analysis, particles were randomly assigned to half-maps for resolution estimation. Randomization method was not utilized to the other experiments because the samples in this study were not allocated into experimental groups during data acquisition and analysis.
Blinding	Blinding is not relevant to this study since the results cannot be affected by whether sample identities were disclosed.

Reporting for specific materials, systems and methods

We require information from authors about some types of materials, experimental systems and methods used in many studies. Here, indicate whether each material, system or method listed is relevant to your study. If you are not sure if a list item applies to your research, read the appropriate section before selecting a response.

Materials & experimental systems

n/a	Involved in the study
☐	☒ Antibodies
☐	☒ Eukaryotic cell lines
☒	☐ Palaeontology and archaeology
☒	☐ Animals and other organisms
☒	☐ Clinical data
☒	☐ Dual use research of concern
☒	☐ Plants

Methods

n/a	Involved in the study
☒	☐ ChIP-seq
☒	☐ Flow cytometry
☒	☐ MRI-based neuroimaging

Antibodies

Antibodies used	anti-FLAG antibody (product number: 20543-1-AP, Proteintech, IL, USA), anti-FTSJ1 antibody (product number: 11620-1-AP, Proteintech, IL, USA) and horseradish peroxidase-conjugated secondary antibodies (product number: 7074, Cell Signaling Technology, MA, USA).
Validation	Each antibody was validated by manufacturer.

Eukaryotic cell lines

Policy information about cell lines and Sex and Gender in Research

Cell line source(s)	Expi293 cells, HEK293FT cells and FreeStyle HEK293F cells were purchased from Themo Fisher.
Authentication	No further authentication was performed to cell lines purchased from Thermo Fisher.
Mycoplasma contamination	Mycoplasma contamination was not tested.
Commonly misidentified lines (See ICLAC register)	No commonly misidentified cell lines were used.

Plants

Seed stocks	None.
Novel plant genotypes	None.
Authentication	None.
